# Exploring the Diversity and Antibacterial Potentiality of Cultivable Actinobacteria from the Soil of the Saxaul Forest in Southern Gobi Desert in Mongolia

**DOI:** 10.3390/microorganisms10050989

**Published:** 2022-05-09

**Authors:** Shao-Wei Liu, Norovsuren Jadambaa, Arina A. Nikandrova, Ilya A. Osterman, Cheng-Hang Sun

**Affiliations:** 1 Department of Microbial Chemistry and Beijing Key Laboratory of Antimicrobial Agents, Institute of Medicinal Biotechnology, Chinese Academy of Medical Sciences & Peking Union Medical College, Beijing 100050, China; liushaowei3535@163.com; 2 The Institute of Biology, Mongolian Academy of Sciences, Ulaanbaatar 13330, Mongolia; norvo@mail.ru; 3 Center of Life Sciences, Skolkovo Institute of Science and Technology, 143028 Moscow, Russia; arinanikandrova@mail.ru (A.A.N.); i.osterman@skoltech.ru (I.A.O.); 4 Department of Soil Science, Lomonosov Moscow State University, 119991 Moscow, Russia; 5 Department of Chemistry, Lomonosov Moscow State University, 119991 Moscow, Russia; 6 Genetics and Life Sciences Research Center, Sirius University of Science and Technology, 354340 Sochi, Russia

**Keywords:** saxaul forest, actinobacteria, diversity, antibacterial metabolites, structural identification, enhanced molecular networking

## Abstract

Saxaul (*Haloxylon ammodendron*) is the most widespread plant community in the Gobi Desert in Mongolia, which plays important roles in wind control, sand fixation and water conservation. Investigations of soil-derived actinobacteria inhabiting in the saxaul forest in Gobi Desert in Mongolia have been scarce. In this study, biodiversity of culturable actinobacteria isolated from soil of the saxaul forest in Southern Gobi Aimak (Southern Gobi Province) of Mongolia was characterized and their potential to produce compounds with antibacterial activities was assessed. A total of 172 actinobacterial strains were recovered by culture-based approaches and were phylogenetically affiliated into 22 genera in 13 families of seven orders. Forty-nine actinobacterial isolates were selected to evaluate the antibacterial activities and their underlying mechanism of action was screened by means of a dual-fluorescent reporter assay (pDualrep2). Twenty-three isolates exhibited antagonistic activity against at least one of the tested pathogens, of which two *Streptomyces* strains can attenuate protein translation by ribosome stalling. Combinational strategies based on modern metabolomics, including bioassay-guided thin-layer chromatography (TLC), UPLC-QTOF-MS/MS based structural annotation and enhanced molecular networking successfully annotated chloramphenicol, althiomycin and granaticin and their derivatives as the antibacterial compounds from extracts in three *Streptomyces* strains, respectively. This work demonstrates that UPLC-MS/MS-based structural identification and enhanced molecular networking are effective strategies to rapidly illuminate the bioactive chemicals in the microbial extracts. Meanwhile, our results show that the saxaul forest in Mongolia Gobi Desert is a prospective source for discovering novel actinobacteria and biologically active compounds.

## 1. Introduction

Antimicrobial resistance (AMR) caused by emergence, persistence and prevalence of multidrug-resistant (MDR) bacteria or “superbugs” can render antibiotics ineffectiveness. It has been creating a significant global threat to public health system [1,2]. It is reported that more than 70% of the pathogenic bacteria are resistant to at least one commonly used antibiotic [3]. Currently, all classes of antibiotics have faced an emergence of resistance that compromises their usage; and it will worsen in the coming decades with continuous failure to spur the development of novel antibiotics and nonjudicious use of existing antibiotics. Therefore, innovative antimicrobial agents with diverse mechanisms of action are still urgently needed [4].

Despite great efforts in chemical synthesis and engineered biosynthesis, natural products, especially microbial metabolites, are still a predominant source of bioactive scaffolds that promote the development of life-saving antibiotics. Actinobacteria isolated from diverse environments are well known for their versatile capabilities to produce chemically diverse and biologically active metabolites, such as antibiotics, immunosuppressants, enzyme inhibitors, antineoplastic and antiviral agents [5,6]. However, depressingly in recent decades, continual rediscovery of known compounds from well-known actinobacterial strains makes a tough access to novel scaffolds with promising activity. Therefore, it is recommended to seek potentially bioactive microorganisms from extreme or untapped environments, such as hyper-arid deserts [7], deep-sea [8,9], invertebrates [10] and volcanics [11]. In recent years, an increasing number of encouraging works achieved from extreme or unexploited environments have demonstrated that traditional approaches of isolating novel microorganisms from unexplored habitats are still promising [12,13,14].

Mongolia is one of the largest land-locked countries in the world, which preserves various and unique ecosystems such as high mountains, taiga forests, permafrosts, steppes, desert-steppes and deserts [15]. Currently, desertification and land degradation in Mongolia have severely affected 90% of the territory, and more than 30% of the territory is occupied by the deserts or semi-deserts [16]. The Gobi Desert in Southern Mongolia has been recognized as the strongest dust storm hot spot [17]. Extremely dry climate, sparse vegetation, intensive ultraviolet radiation, barren soils with poor humus and moisture content make large areas of Gobi Desert in Mongolia pristine and undisturbed [18]. Many research studies have proved an abundant bacterial diversity residing in different regions of the desert zones in Mongolia, and most actinobacterial strains isolated from desert soils or rhizosphere (the environment surrounding plant roots) can produce antagonistic metabolites and proteolytic enzymes [15,19]. Recently, over 800 endophytic strains were isolated from plants growing in the Gobi area of Mongolia by Natsagdorj et al., and their bioactive potentials with anti-quorum sensing and antibacterial activities were explored [20].

Saxaul (*Haloxylon ammodendron*) is the most widespread forest community in the Gobi Desert area in Mongolia. As a startlingly hardy plant, saxaul can survive naturally in the strongest drought, unbearable heat and saline habitats. Saxaul forests play an important role in desert in preventing soil erosion and land degradation, improving sand fixation, water regulation and carbon sequestration [21] and ameliorating the microclimate of ecosphere, thus facilitating the settlement and growth of other desert organisms, including microorganisms [21,22]. Therefore, actinobacteria inhabiting in the saxaul forest of Mongolia Gobi Desert attract our particular interest. To the best of our knowledge, very limited research has been carried out on bioprospecting of soil-derived actinobacteria inhabitant in the saxaul forest in Mongolia, and their associated bioactive substances are hitherto uncharacterized.

In this study, biodiversity of actinobacteria inhabiting in soil of the saxaul forest in the Southern Gobi Desert in Mongolia was investigated by culture-based approaches. Their potential to produce antibacterial compounds against “ESKAPE” [23] pathogens was assessed. Furthermore, a combinational chemical assessment based on modern metabolomics, including bioassay-guided thin-layer chromatography (TLC), UPLC-MS/MS based structural annotation and enhanced molecular networking analysis was carried out on several selected actinobacterial strains. Through this study, we wish to expand our knowledge on the diversity and pharmaceutical potential of actinobacteria residing in this region and demonstrate Mongolia desert as one of special environments deserves more bioprospecting studies.

## 2. Materials and Methods

### 2.1. Samples Collection and Preparation

**Figure 1:**
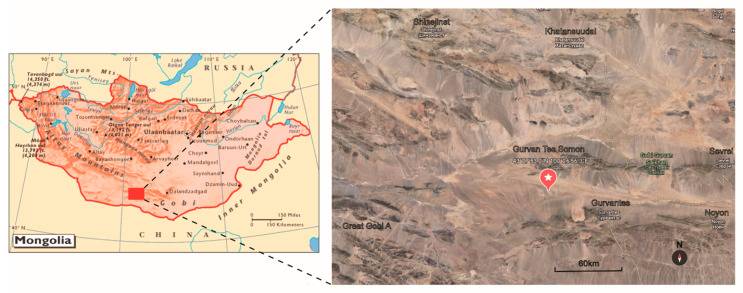
Sampling site. The map shows the sample collection site in Gurvan Tes Somon (Gurvan Tes County) in the Southern Gobi Aimak (Southern Gobi Province) in Mongolia.

Soil samples were collected in a saxaul forest in Gurvan Tes Somon (Gurvan Tes County), located in the Southern Gobi Aimak (Southern Gobi Province) in Mongolia (43.46479° N, 100.44897° E) at an altitude of 1502 m (Figure 1). The soil samples were collected at a depth of 5–15 cm from the dried soil surface, and then packed into 50 mL sterile Falcon tubes. Samples were air-dried at room temperature for 8 h in the laminar flow hood before isolation.

### 2.2. Actinobacteria Isolation

The soil samples were processed by using the serial dilution plating technique. Briefly, 3 g of soil sample was suspended in 30 mL of sterile physiological saline (NaCl, 9 g/L) and shaken on a rotatory shaker at 180 rpm for 2 h. The soil suspension was serially ten-fold diluted to 10^−3^, and 0.2 mL of the dilutions (10^−2^ and 10^−3^) from each soil sample were spread onto different isolation media. Ten selective media supplemented with 1% (*v*/*v*) soil leaching liquor were prepared to isolate the actinobacterial strains (Supplementary Table S1). The soil leaching liquor was prepared as follows: 200 g dry soil was added into 200 mL distilled water and then boiled for 30 min. After cooling down, the soil soup was filtered by absorbent cottons to obtain the soil leaching liquor. All media were supplemented with nalidixic acid (20 µg/mL in final concentration), cycloheximide (50 µg/mL) and potassium dichromate (50 µg/mL) as inhibitors of the fast-growing Gram-negative bacteria and fungi. After incubation at 30 °C for 2–8 weeks, colonies that displayed different morphologies were picked up and then individually sub-cultured on the International *Streptomyces* Projects 2 (ISP 2) [24] agar plates until pure colonies were obtained. Purified isolates were maintained on ISP 2 agar slants at 4 °C and preserved in 20% (*v*/*v*) glycerol suspensions at −80 °C.

### 2.3. Taxonomic Identification of the Obtained Isolates

Primary taxonomic identification of all isolates was performed on the basis of 16S rRNA gene sequencing. Genomic DNAs were extracted as described previously with the Chelex-100 reagent [25]. The 16S rRNA genes were amplified by PCR with two universal primers 27F (5′-AGAGTTTGATCMTGGCTCAG-3′) and 1492R (5′-GGTTACCTTGTTACGACTT-3′). The PCR mixture (30 µL) contained 15 µL of 2 × PCR Supermix (TransGen Biotech, Beijing, China), 0.9 µL of each primer (10 mM, Sangon Biotech, Shanghai, China), 0.9 µL of genomic DNA and 12.3 µL of nuclease-free water. The amplified products were sent to Shanghai Sangon Biotech Company for purification and sequencing. The taxonomic affiliation of the strains was determined using the BLAST tool in GenBank NCBI database (http://www.ncbi.nlm.nih.gov/ (accessed from 10 March 2019 to 25 June 2019)) and the EzBioCloud Identify service (https://www.ezbiocloud.net/identify (accessed from 10 March 2019 to 25 June 2019)) [26]. According to BLAST results, the corresponding sequences of closely related type species were retrieved from the GenBank database, and multiple alignments were aligned using the ClustalW tool in MEGA 11 (version 11.0.10, Tamura, K. et al., Philadelphia, PA, USA) [27]. A phylogenetic tree was then constructed using the neighbor-joining algorithm [28] based on the Kimura’s two-parameter model [29] with 1000 bootstrap replicates. The 16S rRNA gene sequences obtained in this study were deposited in GenBank under the accession numbers OM943700-OM943745.

### 2.4. Extracts Preparation and Antimicrobial Assay

For assessment of antibacterial potential of the isolated actinobacterial strains, 49 out of 172 isolates affiliated to 22 genera were selected to conduct a small-scale fermentation. The 49 different strains were selected on the basis of phylogenetic analyses of partial 16S rRNA gene sequences, as well as comparison of morphological characteristics, such as colonial morphology and diffusible pigments. Strains were cultivated in Erlenmeyer flasks (500 mL) containing of 100 mL ISP2 medium or Tryptone Soya Broth (TSB). The cultures were incubated at 30 °C under 180 rpm for 3–14 days, depending on the proliferation rate of each strain under the offered conditions. The cultural liquid and mycelia were separated by centrifugation at 4500 rpm for 20 min. Metabolites from the supernatant were extracted twice by using a separatory funnel with an equal volume of ethyl acetate (EA, 100 mL). The upper organic layer was condensed by rotary evaporation under reduced pressure, and then dissolved in 1 mL methanol to obtain the crude extract. The water layer (60 mL) was lyophilized and then dissolved in 1 mL of 50% methanol/water solution. The mycelia were soaked overnight in acetone, and then the leach liquor was concentrated under vacuum and residues were dissolved in 1 mL of 50% methanol/water solution. Consequently, three types of samples (EA extract, water layer extract and mycelia extract) from each strain were ready for antimicrobial assay.

Antibacterial activities of three types of samples were evaluated by paper-disk diffusion method. Specifically, 50 μL tested sample was dripped on a sterile paper disk (diameter, 6 mm) and dried up under ambient temperature, then the paper disk was placed on the MH (Mueller-Hinton) agar containing the indicator strains. Fifty microliters of pure methanol as the blank control and ten microliters (100 μg/mL) of levofloxacin as the positive control were also tested parallelly in each plate. Antibacterial activity was evaluated by measuring the inhibitory halos after the agar plates incubated at 37 °C for 18 h. Twelve “ESKAPE” strains including *Enterococcus faecalis* (ATCC 33186 and NO. 310682), *Staphyloccocus aureus* (ATCC 29213 and ATCC 33591), *Klebsiella peneumoniae* (ATCC 10031 and ATCC 700603), *Acinetobacter baumannii* (NO. 2799 and ATCC 19606), *Pseudomonas aeruginosa* (ATCC 27853 and NO. 2774) and *Escherichia coli* (ATCC 25922 and ATCC 35218) were used as indicator pathogens. All strains mentioned above were deposited in the Institute of Medicinal Biotechnology, Chinese Academy of Medical Sciences.

### 2.5. Determination of Antibacterial Mechanism

A dual-fluorescent reporter system with the reporter strain “JW5503-pDualrep2” was used to probe the inhibitors of protein and DNA biosynthesis as described previously [30]. In brief, 100 μL ethyl acetate extract of each strain was dried up and then dissolved in 100 μL DMSO. About 2 μL of the DMSO solution was spotted on the agar plate containing a lawn of the reporter strain *E. coli* JW5503 Δ*tolC*, which was transformed by the reporter plasmid “pDualrep2”. After overnight incubation at 37 °C, the plate was scanned by the ChemiDoc Imaging System (Bio-Rad Laboratories, Hercules, CA, USA) with two channels, “Cy3-blot” (553/574 nm, green pseudocolor) for red fluorescent protein (RFP) fluorescence, and “Cy5-blot” (588/633 nm, red pseudocolor) for Katushka2S fluorescence. Induction of expression of Katushka2S is triggered by translation inhibitors, while RFP is upregulated by DNA damage-induced SOS response. Erythromycin (Ery, 5 mg/mL, 1 μL) and Levofloxacin (Lev, 50 μg/mL, 1 μL) were used as positive controls for inhibitors of protein and DNA biosynthesis, respectively.

### 2.6. Bioassay-Guided Thin-Layer Chromatography (TLC)

Ethyl acetate extracts from the cultural broth of three actinobacterial strains MGa1-3, MGa2-5 and MGa5-5 were analyzed by TLC. One hundred microliters of crude EA extracts were automatically loaded onto a 10 cm × 10 cm precoated Silica gel 60 F_254_ TLC plate (Merck, Darmstadt, Germany) by using a semi-automatic sample applicator Linomat 5 (CAMAG, Muttenz, Switzerland) with a 500 µL Hamilton syringe. The sampled TLC plates were developed by using 10 mL methanol/dichloromethane (*v*/*v*, 1/9) as the developing reagent. The plates were visualized under 254 nm and 366 nm with the CAMAG TLC Visualizer. A piece of TLC plate (10 cm × 1 cm) was cut off and then stuck on an agar plate containing a lawn of methicillin-resistant *Staphylococcus aureus* (MRSA) ATCC 33591 as the indicator strain. After overnight incubation at 37 °C, bioactive TLC bands were scraped and then eluted with methanol for the further UPLC-UV-HRMS/MS analysis.

### 2.7. UPLC-QTOF-MS/MS Analysis

EA extracts from the cultural broth or methanol eluates of the bioactive TLC bands were dried up and redissolved in an appropriate volume of LC/MS-grade methanol to the final concentration of 1 mg/mL, and then analyzed by the UPLC-QTOF-MS/MS system (ACQUITY UPLC/Xevo G2-XS QTOF, Waters, Milford, MA, USA) equipped with an electrospray ionization source (ESI). One microliter of each extract was injected into a Waters ACQUITY UPLC BEH C18 column (2.1 × 100 mm, 1.7 μm). Chromatographic separation was carried out at a flow rate of 0.3 mL/min by using 99.9% water/0.1% formic acid (LC/MS grade) as the mobile phase A and 100% acetonitrile (LC/MS grade) as the mobile phase B. A linear gradient of phase B from 10% to 90% for 18 min followed by 90% B for 2 min before returning to the initial condition was employed.

Mass spectra were acquired in two modes: data-independent acquisition mode (MS^E^) can provide MS/MS fragmentation of precursor ions without ions selection, which was used for compounds dereplication in the UNIFI platform (Waters, United States) [31]; data-dependent acquisition mode (DDA) enabled automatic intensity-ranking of precursor-triggered MS/MS fragmentation, which was used for construction of the Global Natural Products Social (GNPS) Molecular Network. Mass detection was performed in the positive mode with the following parameters: source temperature, 100 °C; desolvation temperature, 250 °C; sampling cone voltage, 40 eV; capillary voltage, 2 kV; source offset voltage, 80 eV; cone gas, 30 L/h; and desolvation gas, 600 L/h. MS^E^ detection was performed in continuum format with the mass range from 100 to 1500 Da in low collision-energy (CE) of 2 V and a high ramp CE of 40–60 V, and the scan time was 0.2 s.

The DDA scan was performed almost the same as the MS^E^ mode, except for the following settings: the dual-dynamic collision energy was set from 20–40 V for low-mass collision energy (LM CE), and 60–80 V for high-mass collision energy (HM CE). Automatic switching to MS/MS acquisition occurred when the TIC intensity of an individual ion rose above 10,000 counts and was switched off when accumulated intensity over 1,000,000 counts or when 0.4 s had elapsed. The full MS survey was performed in the range of 100–1500 Da for 0.2 s, and MS/MS scanned with a mass range of 50–1500 Da by the same scan time. Five ions with the highest intensity were automatically selected further for MS/MS fragmentation spectra. Data were collected and analyzed with the MassLynx V4.1 software (Waters, United States). As controls, blank solvent (methanol) and extract of non-inoculated medium were analyzed under the same conditions.

### 2.8. Molecular Networking and Dereplication Analysis

LC-MS/MS raw data in DDA format of strains MGa5-5, MGa2-5 and MGa1-3 obtained were converted to the mzML format using MSConvert [32]. A Classical molecular network was generated using the GNPS online workflow (http://gnps.ucsd.edu (accessed on 23 April 2021)) [33]. Related parameters were set as follows: minimum cosine score, 0.6; parent mass tolerance, 0.1 Da; fragment ion tolerance, 0.1 Da; network topK, 10; minimum matched peaks, 4; maximum cluster size, 100; minimum cluster size, 2. Data visualization of the molecular network was performed on Cytoscape 3.9.1 [34]. The spectra in the network were searched against GNPS spectral libraries for initial dereplicating. Subsequently, Network Annotation Propagation (NAP) in GNPS was used to conduct in silico structural annotation [35]. The GNPS, Super Natural II (SUPNAT), Chemical Entities of Biological Interest (ChEBI) and DRUGBANK structural databases were searched in NAP. The in silico annotations given by NAP were integrated into the generated molecular network by MolNetEnhancer in GNPS [36]. The predicted structures in the Enhanced molecular network were visualized in Cytoscape using the chemViz2 plugin [34]. Manual annotation of metabolites was conducted in UNIFI data processing workflow by searching the microbial natural products databases, The Natural Product Atlas (NPAtlas, www.npatlas.org (accessed on 12 May 2021)) [37] and StreptomeDB [38]. Structures of compounds 1 (granaticin A) and 5 (chloramphenicol) were confirmed by comparisons of the chromatographic and MS spectrum with the authentic standards, while others by the interpretation of MS data, MS/MS fragmentation and comparison with the related literatures when the standards were not available.

## 3. Results

### 3.1. Biodiversity and Phylogenetic Novelty of Cultivable Actinobacteria

**Figure 2:**
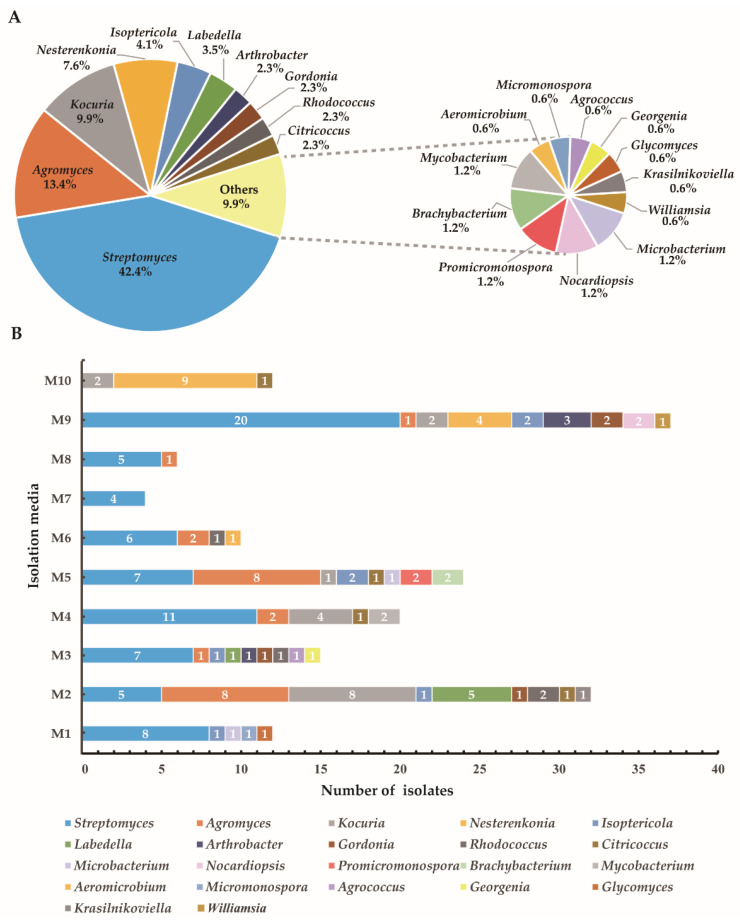
Diversity of cultivable actinobacteria recovered from the soil of the saxaul forest in the Southern Gobi Aimak in Mongolia. (**A**) Pie chart showing the percentage of recovered isolates affiliated with 22 different genera. (**B**) Numbers of actinobacteria in different genera isolated using ten selective culture media (M1–M10). Compositions of the ten-culture media (M1–M10) are shown in Supplementary Table S1.

Overall, 172 actinobacterial strains were obtained from the soil samples collected in the saxaul forest in Southern Gobi Aimak in Mongolia. The isolates belonged to seven orders: *Micrococcales* (48.3%, 83 strains), *Streptomycetales* (42.4%, 73 strains), *Mycobacteriales* (6.4%, 11 strains), *Streptosporangiales* (1.2%, 2 strains), *Glycomycetales* (0.6%, 1 strain), *Micromonosporales* (0.6%, 1 strain) and *Propionibacteriales* (0.6%, 1 strain); comprising 22 genera in 13 different families (Supplementary Table S2). Relative abundances of the isolates at the genus level (Figure 2A) indicated that most abundant genus was *Streptomyces* (42.4%, 73 strains), followed by *Agromyces* (13.4%, 23 strains), *Kocuria* (9.9%, 17 strains), *Nesterenkonia* (7.6%, 13 strains), *Isoptericola* (4.1%, 7 strains) and *Labedella* (3.5%, 6 strains). Additionally, seven genera including *Aeromicrobium*, *Micromonospora*, *Agrococcus*, *Georgenia*, *Glycomyces*, *Krasilnikoviella* and *Williamsia* were recovered by only one isolate. The selective media exerted a major influence on the diversity of isolates obtained (Figure 2B). The Casein-Glucose medium (M9) appeared most effective regarding the number and diversity of isolates obtained (37 strains distributed in 9 genera). The ISP2 medium (M2) yielded the second-highest recovery effect with 32 isolates distributed in 9 genera recovered. The Trehalose–Proline medium (M7) yielded the lowest recovery effect with four strains distributed in one genus *Streptomyces*. The high-salt medium (M10) was used to isolate halophilic or halotolerant strains, and 12 strains belonged to three genera (*Nesterenkonia*, *Kocuria* and *Citricoccus*) were isolated from this medium. Noteworthily, genus *Nesterenkonia* were isolated only from M9 and M10, which were added with 8% and 16% (*w*/*v*) multi-salts, respectively. Genera *Promicromonospora* and *Brachybacterium* were isolated only from CMKA [39] medium (M5).

Based on BLASTN analysis of 16S rRNA gene sequences, five strains exhibited relatively low sequence similarities (<98.65%, the threshold for species delineation [40]) with validly published species, indicating these isolates might represent novel taxon in the phylum *Actinobacteria*. Phylogenetic analysis based on almost complete 16S rRNA gene sequences (>1300 bp) showed these potential novel strains belonged to four genera, including strains MGc2-7 and MGb5-5 in genus *Brachybacterium* of family *Dermabacteraceae*, strain MGb5-11 in genus *Agromyces* of family *Microbacteriaceae*, strain MGc9-5 in genus *Nocardiopsis* of family *Nocardiopsaceae* and strain MGc1-11 in genus *Glyomyces* of family *Glycomycetaceae*. The 16S rRNA gene sequences of strain MGb5-5, MGc2-7, MGc9-5, MGb5-11 and MGc1-11 shared highest similarities with *Brachybacterium sacelli* LMG 20345^T^ (98.4%), *Brachybacterium sacelli* LMG 20345^T^ (98.4%), *Nocardiopsis halotolerans* DSM 44410^T^ (98.2%), *Agromyces kandeliae* Q22^T^ (98.3%) and *Glycomyces harbinensis* IFO14487^T^ (98.5%), respectively. Further phylogenetic analysis based on the neighbor-joining algorithm is shown in Supplementary Figure S1. These putative novel species will be further identified by the polyphasic approach to determine their taxonomic positions.

### 3.2. Assays of Antimicrobial Activity and Mechanism Action

**Figure 3:**
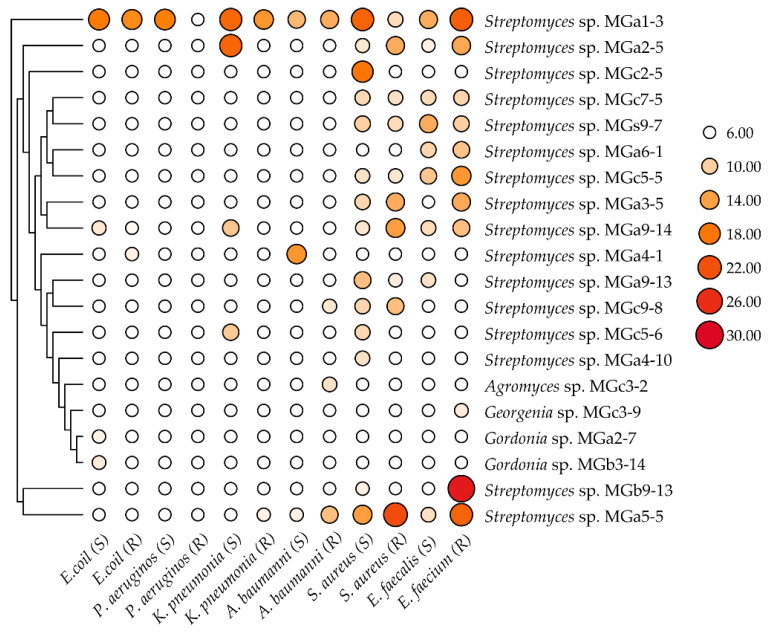
Antibacterial activity of bioactive ethyl acetate extracts from cultural broth of 20 strains. Numbers alongside the circles represent the diameter (mm) of the inhibition halos. 6.00 mm, no inhibitory activity; S, drug-sensitive; R, drug-resistant.

Bioassay is generally the premier step to discover novel antibiotics. Based on morphological comparison and phylogenetic analyses, 49 actinobacterial strains affiliated to 22 different genera were selected to investigate their antimicrobial activities against a panel of “ESKAPE” strains. Out of the 49 strains, 23 exhibited antagonistic activity against at least one of the tested “ESKAPE” pathogens. These bioactive strains were affiliated to five genera including *Streptomyces* (18 strains), *Gordonia* (2), *Georgenia* (1), *Agromyces* (1) and *Mycobacterium* (1). The antibacterial spectra of the 23 isolates against different indicator bacteria are shown in Supplementary Table S3. Among the 23 antimicrobial isolates, eleven isolates showed inhibitory activity only against Gram-positive bacteria; four isolates exhibited antagonistic activity only against Gram-negative bacteria; and eight isolates against both Gram-positive and Gram-negative bacteria. The majority of strains (20 strains) demonstrated antibacterial activity in the EA extracts from their cultural broth, and the antibacterial spectra of EA extracts from the 20 strains are displayed in Figure 3. Three strains, including *Mycobacterium* sp. MGb4-1, *Streptomyces* sp. MGb9-6 and *Streptomyces* sp. MGc3-10 only showed antibacterial activities from the extracts of the water phase. Three *Streptomyces* isolates including MGa9-14, MGa5-5 and MGa1-3, showed broader spectrum of antibacterial activity against at least seven indicator bacteria.

**Figure 4:**
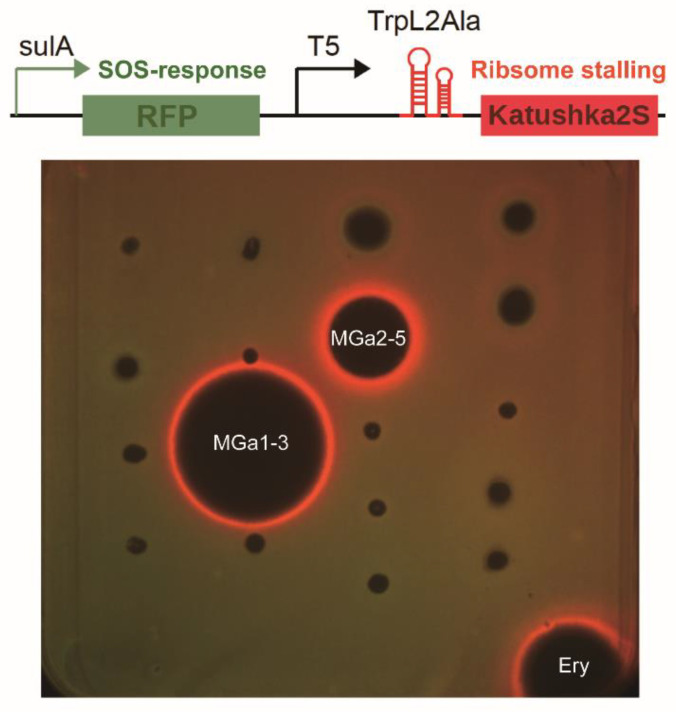
The dual-fluorescent reporter “pDualrep2” system sensitive to inhibitors of ribosome progression or DNA replication. Spots of erythromycin (Ery) and test samples were placed on an agar plate coated with a layer of *E. coli* JW5503 Δ*tolC* strain that was transformed with the pDualrep2 plasmid. Induction of the expression of Katushka2S is triggered by translation inhibitors, whereas RFP expression occurs on DNA-damage induced SOS-response. The plate was scanned under the ChemiDoc Imaging System at 553/574 nm and 588/633 nm channels to detect RFP (green pseudocolor) and Katushka2S (red pseudocolor) fluorescence, respectively.

To distinguish antibacterial mechanisms of the 23 bioactive strains, EA extracts of cultural broth were screened by a unique high-throughput screening model based on a dual-fluorescent reporter “pDualrep2” system (Figure 4). The reporter strain “JW5503-pDualrep2” was constructed with an *E. coli* strain JW5503 (Δ*tolC*) transformed with the pDualrep2 plasmid as described previously [30]. In this reporter system, expression of RFP gene (shown in green pseudo-color) is under regulation of *sulA* promoter and activated when compounds induce a SOS response to the DNA damage. Meanwhile, Katushka2S protein is expressed (far-red fluorescent) in the presence of translation inhibitors, which trigger ribosome stalling at the mutated leader peptide (trpL-2Ala) of a tryptophan operon. The “pDualrep2” system has been proved to be a highly sensitive biosensor for probe of compounds that attenuates protein translation or DNA biosynthesis. In this study, extracts recovered from the two *Streptomyces* strains, MGa1-3 and MGa2-5, induced expression of far-RFP reporter Katushka2S, acting as typical inhibitors of protein biosynthesis due to the translation stalling on ribosome, similar to erythromycin. However, no hit was found to induce expression of RFP reporter, suggesting no strain could produce compounds triggering DNA damage as the example of levofloxacin (Figure 4).

### 3.3. Chemical Identification of Extracts from Strains MGa1-3, MGa2-5 and MGa5-5

**Figure 5:**
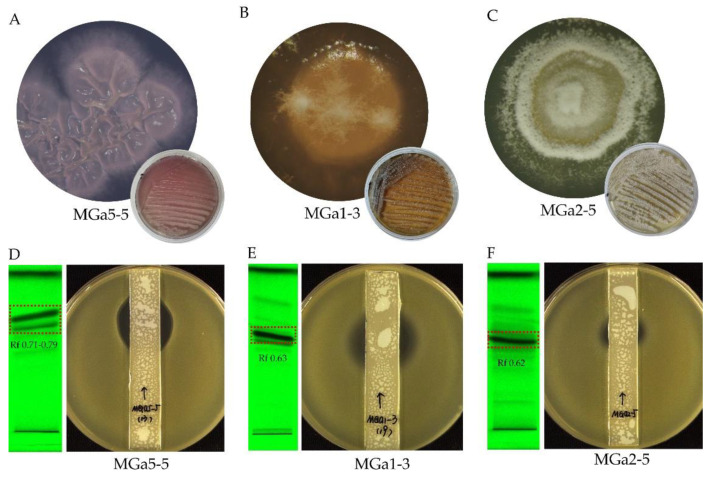
Colony morphology of *Streptomyces* isolates MGa5-5 (**A**), MGa1-3 (**B**) and MGa2-5 (**C**), and TLC analysis of the EA extracts from fermentation broth (**D**–**F**). Colony morphology was photographed after growing on ISP2 media for about 7 days. TLC analysis was coupled with anti-MRSA assay, and TLC bands was visualized at 254 nm.

To gain a fast survey of the nature of chemicals possibly contributing to the antibacterial activity, we conducted TLC analysis followed by UPLC-MS/MS based structural annotation for the secondary metabolites produced by three *Streptomyces* strains, MGa1-3, MGa2-5 and MGa5-5, which exhibited strong antagonistic activities against the tested “ESKAPE” pathogens. Furthermore, strain MGa1-3 and MGa2-5 displayed translation inhibitory action in the pDualrep2 assay (Figure 4), and strain MGa5-5 produced a special diffusible violet-blue pigment in ISP2 medium (Figure 5A).

BLASTN analysis based on the 16S rRNA sequences of strains MGa5-5, MGa1-3 and MGa2-5 suggested that all these strains belonged to the genus *Streptomyces*. The nearly full-length 16S rRNA gene sequence of strain MGa5-5 (GenBank NO. OM943740, 1396 bp) shared the highest sequence similarity (99.7%) with *S. lateritius* LMG 19372^T^. Strain MGa1-3 (GenBank NO. OM943745, 1396 bp) showed the highest identity (99.7%) to *S. venezuelae* ATCC 10712^T^, and strain MGa2-5 (GenBank NO. OM943743, 1399 bp) shared a maximum sequence identity (98.8%) with *S. althioticus* NRRL B-3981^T^.

EA extracts from strains MGa1-3, MGa2-5 and MGa5-5 were investigated by TLC to assess the composition of secondary metabolites. The extracts displayed some distinct bands under 254 nm (Figure 5D–F). Antibacterial assay of the TLC plates against methicillin-resistant *Staphylococcus aureus* showed the active bands (R_f_ 0.63 for strain MGa1-3, R_f_ 0.62 for strain MGa2-5 and R_f_ 0.71–0.79 for strain MGa5-5) as illustrated in Figure 5D–F. Components in these active bands were scraped, eluted with methanol and further analyzed by UPLC-UV-QTOF MS/MS.

**Figure 6:**
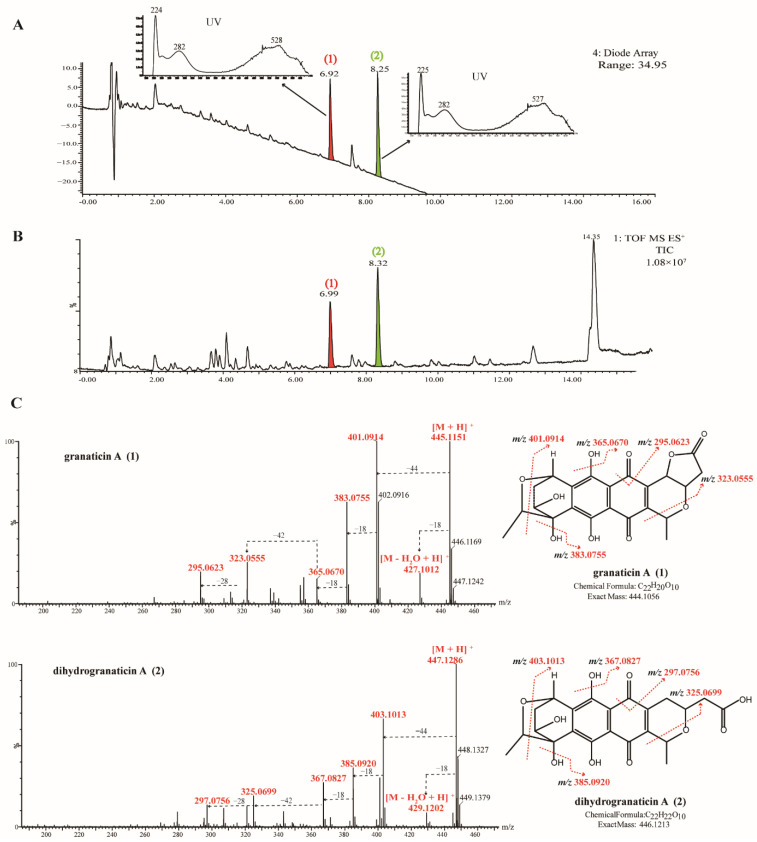
UPLC-UV-HRMS/MS spectra and identification of bioactive compounds from EA extract of strain MGa5-5. (**A**) UPLC-UV chromatogram at 280 nm of the bioactive TLC band (R_f_ 0.71–0.79) extracted from strain MGa5-5, and two major compounds (peak **1** and **2**) were labelled in the UPLC spectrum. (**B**) Total ion chromatogram (TIC) of the bioactive TLC band (R_f_ 0.71–0.79) extracted from strain MGa5-5. (**C**) MS/MS spectra and the annotated fragmentation patterns of peak **1** (granaticin A) and **2** (dihydrogranaticin A).

The UV spectra, molecular formulae and accurate masses from UPLC-UV-HRMS/MS analysis were searched against NPAtlas and StreptomeDB database in the UNIFI informatics platform to identify possible compounds. UPLC-UV-HRMS/MS profile of the active TLC band (R_f_ 0.71–0.79) extracted from strain MGa5-5 is shown in Figure 6. The total ion chromatogram (TIC) of strain MGa5-5 exhibited two predominant peaks at retention time (tR) 6.99 min (Peak **1**) and 8.32 min (Peak **2**), with the molecular ion of 445.1151 [M + H]^+^ and 447.1286 [M + H]^+^, respectively. The two components were putatively annotated as two granaticin derivatives, granaticin A (**1**) and dihydrogranaticin A (**2**). The retention time, UV absorption and specific MS/MS fragmentation of peak **1** (granaticin A) matched perfectly with that of the authentic standard (Supplementary Figure S2). Granaticin is a member of aromatic polyketide antibiotics known as the benzoisochromanequinones (BIQs). It is structurally quite similar to actinorhodin, but with a opposite stereochemistry of the pyran ring and a unique 2,6-dideoxysugar annealed to the aromatic ring system [41,42,43]. It is reported that granaticin A exhibited antibacterial activities against Gram-positive bacteria and cytotoxic activities against some cancer cell lines, but have little or no effect on Gram-negative bacteria [44,45]. Dihydrogranaticin A has similar antibacterial spectrum with granaticin A [43,46].

In the sample eluted from the active TLC band (R_f_ 0.62) of *Streptomyces* sp. MGa2-5, a prominent peak observed at 5.09 min (Peak **3**) with the monoisotopic weight of 440.0713 [M + H]^+^, was putatively annotated as a pentapeptide antibiotic, althiomycin (**3**, Supplementary Figure S3). The result was supported by comparison of the precursor ion *m/z* value, fragmentation pattern and UV spectrum with reference data [47,48,49]. Althiomycin is a sulfur-containing cyclic peptide discovered from strains of *Streptomyces* [47,48], *Myxococcus* [50], *Cystobacter* [51] and *Serratia* [52], and it is reported to be effective against both Gram-positive and Gram-negative bacteria and possess low toxicity [53,54,55].

In the same manner, active components appearing in the TLC band (R_f_ 0.63) extracted from strain MGa1-3 were analyzed (Supplementary Figure S4). Two prominent peaks observed at 5.17 min (Peak **4**) and 6.12 min (Peak **5**), with the monoisotopic weights of 283.1319 [M + H]^+^ and 323.0188 [M + H]^+^, were putatively identified as corynecin III (**4**) and chloramphenicol (**5**), respectively. Both compound **4** and **5** showed characteristic UV absorption of chloramphenicol family at about 278 nm [56], and the structure of compound **5** was unambiguously confirmed by chromatographic and MS spectrum comparisons with the authentic sample of chloramphenicol (Supplementary Figure S5). Chemical structure of corynecin III is similar to chloramphenicol, only differing in lacking two chlorines in the acyl group [57]. In addition to *Streptomyces* strains, a series of corynecin complex (corynecin I, II, III, IV and V) were also isolated exclusively from culture broth of *Corynebacterium hydrocarboclastus*. Corynecin III exhibits a broad antibacterial spectrum similar to chloramphenicol, but is less potent against both Gram-positive and Gram-negative bacteria [57,58].

### 3.4. Enhanced Molecular Networking Analysis

**Figure 7:**
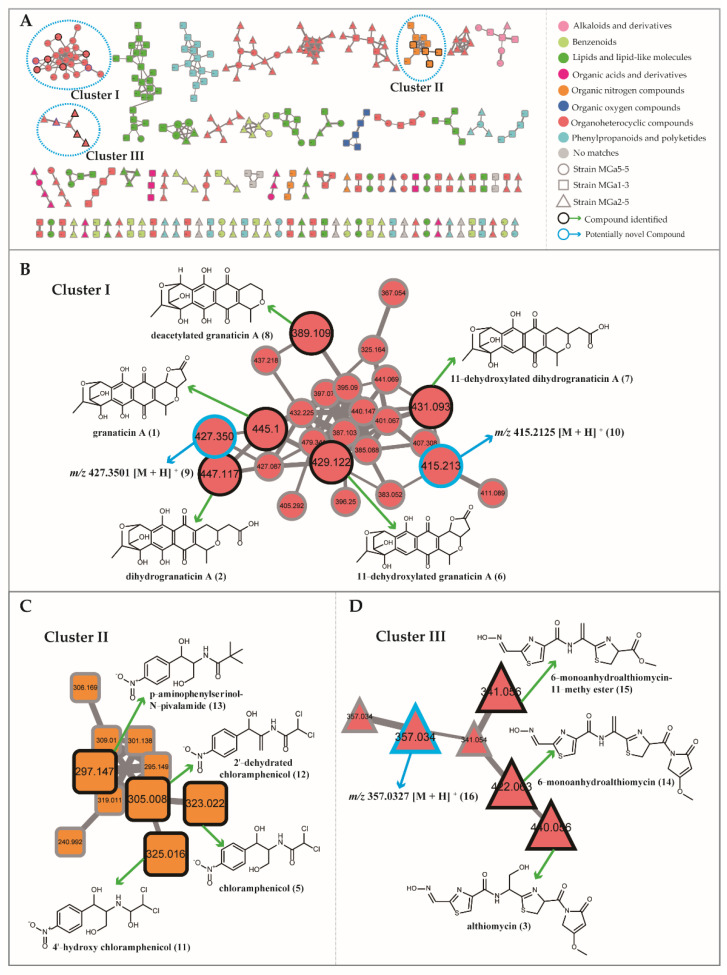
Enhanced molecular network for annotation of secondary metabolites from EA extracts from strains MGa5-5, MGa2-5 and MGa1-3. The molecular network generated by LC-MS/MS data was analyzed by using NAP and MolNetEnhancer workflow via the GNPS platform. (**A**) Structural annotation for the whole molecular families of three strains, wherein the color of nodes denotes the structural annotation at the superclass level by NAP. (**B**) Observation of molecular cluster I allows highlighting annotation of granaticin family in strain MGa5-5. (**C**) Observation of molecular cluster II allows highlighting annotation of chloramphenicol family in strain MGa1-3. (**D**) Observation of molecular cluster III allows highlighting annotation of althiomycin family in strain MGa2-5.

For a comprehensive chemical overview of strains MGa5-5, MGa2-5 and MGa1-3, a molecular network based on MS/MS data from extracts of three *Streptomyces* was generated using the GNPS online platform. After removal of nodes associated with the solvent blank and growth medium blank, a total of 2455 precursor ions were organized into a molecular network, in which 258 were grouped into 94 clusters (nodes ≥ 2). It was noted that not each network node corresponded to a single molecule, since some nodes represented different adducts or charges of the same compounds [59]. To maximize annotation coverage over the entire metabolomics data, we employed MolNetEnhancer, a recently developed workflow that integrated metabolome mining and annotation tools (such as Network Annotation Propagation, MS2LDA or DEREPLICATOR) into molecular networks [36]. Herein, an in silico annotation tool Network Annotation Propagation (NAP) was used to sign a consensus candidate structure for each fragmentation spectrum, then clusters or molecular families (with more than two nodes) were automatically chemically classified through the ClassyFire tool [60]. In the enhanced molecular network (Figure 7A), a total of eight types of chemical family were annotated at the superclass level, and the organoheterocyclic compounds occupied the maximum proportion. Through elaborate inspection of molecular networking annotations, three clusters (Cluster I, II and III) were of particular interest due to the presence of precursor mass matching with the identified antibiotics. Clusters I, II and III separately corresponded to granaticins in strain MGa5-5, chloramphenicol derivatives in strain MGa1-3 and althiomycins in strain MGa2-5 (Figure 7B–D).

The molecular network of the granaticin family (Cluster I, Figure 7B) contained 25 nodes related to different precursor masses ranging from *m/z* 325 to 479 Da. Besides the identified compounds granaticin A (**1**) and dihydrogranaticin A (**2**), three derivatives (**6**, **7** and **8**) corresponding to the 11-dehydroxylated granaticin A (*m/z* [M + H]^+^ 429.1202, C_22_H_21_O_9_, compound **6**), 11-dehydroxylated dihydrogranaticin A (*m/z* [M + H]^+^ 431.0920, C_22_H_23_O_9_, compound **7**) and deacetylated granaticin A (*m/z* [M + H]^+^ 389.1099, C_20_H_21_O_8_, compound **8**) were tentatively identified based on their accurate masses, fragmentation patterns and comparison with the related literatures [61,62,63] (Supplementary Figure S6). Compound **9** showed a [M + H]^+^ ion at *m/z* 427.3501 was inferred as dehydro-granaticin A on the basis of the loss of H_2_O (18 Da) from granaticin A. Compound **10** ([M + H]^+^ 415.2125) was predicted to be a deoxidized analogue of compound **7** based on the molecular formula difference (16 Da). No matches were found in the database to compounds **9** and **10**, suggesting that they could be novel derivatives of granaticins.

The chloramphenicol cluster (Cluster II, Figure 7C) contained 10 nodes with different precursor masses ranging from *m/z* 241 to 323 Da. Starting with the chloramphenicol (**5**), three additional chloramphenicol derivatives were identified through the propagation of annotation of connected nodes, which included 4′-hydroxy chloramphenicol (**11**, *m/z* [M + H]^+^ 325.0153, C_11_H_14_C_l2_N_2_O_5_) [64], 2′-dehydrated chloramphenicol (**12**, *m/z* [M + H]^+^ 305.0081, C_11_H_11_Cl_2_N_2_O_4_) [65] and *p*-aminophenylserinol-*N*-pivalamide (**13**, *m/z* [M + H]^+^ 297.1461, C_14_H_21_N_2_O_5_) [66]. UPLC-MS/MS spectra of these putative chloramphenicol derivatives (**11**–**13**) were illustrated in Supplementary Figure S7. In the molecular network of the althiomycin family (Cluster III, Figure 7D), the protonated molecular ion of althiomycin (**3**) was clustered with five additional nodes. Compound **14** (*m/z* [M + H]^+^ 422.0600, C_16_H_16_N_5_O_5_S_2_) directly connected to the node of althiomycin was unambiguous identified as 6-monoanhydroalthiomycin [67]. Compound **15** protonated molecular ion at *m/z* [M + H]^+^ 341.0509 with the formula C_12_H_13_N_4_O_4_S_2_ was annotated as 6-monoanhydroalthiomycin-11-methy ester [48]. Compound **16** with [M + H]^+^ ion at *m/z* 357.0327 was directly connected to **15**, and the mass difference of 16 Da between two nodes corresponded to the neutral loss of an oxygen. This node could not be annotated, neither by database search nor by fragmentation pattern, suggesting that it may be a new althiomycin analog, though further investigations are required to ascertain it. UPLC-MS/MS spectra of these putative althiomycin derivatives (**14**–**16**) were illustrated in Supplementary Figure S8.

## 4. Discussion

A few published studies have demonstrated that actinobacteria constitute a significant portion in Mongolian microbial population. Incidence of actinobacteria in the desert soils is almost equal to that in the chestnut soils of the steppe zones in Mongolia [15]. Much higher abundance of actinobacterial species has been revealed in the Mongolia desert compared with that of the Atacama Desert [68,69]. This phenomenon may be attributed to the relatively richer organic matters, higher annual rainfall and larger amounts of vegetation in Mongolia desert, resulting in more favorable conditions of aeration, water exchange and nutrient supply [70,71]. A previous study performed by our research group to investigate the diversity of the actinobacteria isolated from the dried beds soils of temporary rivers in the saxaul forest in Mongolia has showed that the total number of actinobacterial strains was about 3.0 × 10^3^–3.2 × 10^4^ CFU/g of soil, whereas only 10 *Streptomyces* strains were isolated and identified [72]. In the present study, 172 actinobacterial strains assigned to 22 genera in 13 families of 7 orders were retrieved from soil of the saxaul forest in the Southern Gobi of Mongolia, with the most isolates affiliated with the genera *Streptomyces*, *Agromyces*, *Kocuria* and *Nesterenkonia*. Notably, genera *Citricoccus, Georgenia, Gordonia and Krasilnikoviella* were registered in Mongolia for the first time. The genus *Streptomyces* was the most widespread in soil of Mongolia saxaul forest, which is congruent with several published studies on actinobacterial communities of Mongolia desert region [15,70,71,72,73]. This is not surprising, since many *Streptomyces* species can produce dormant uninucleoid spores (such as exospores or arthrospores) to minimize metabolism in *Streptomyces* strains to survive in harsh environments, such as the high temperature, desiccation and intense ultraviolet radiation [74].

Utilization of various selective media is quite essential to enhance the bacterial biodiversity in the isolation process. In this study, ten different culture media were applied, leading to recovery of 22 actinobacterial genera and five potential novel species. Casein-Glucose (M9), ISP2 (M2), CMKA (M5) and Modified Cellulose–Casein (M4) media showed the highest recovery rate of actinobacteria. Raffinose-Histidine (M6) medium has been previously described as an efficient medium for the isolation of novel *Streptomyces* species from deserts [75], whereas it did not present an advantage in our study and only allowed retrieval of ten actinobacterial strains. Addition of osmo-protectant substances, proline and trehalose, in Trehalose–Proline medium (M7), was used to enhance the microbial antioxidative defense system against salinity stress [76], but herein only four *Streptomyces* strains were obtained from the medium. This could be explained by the fact that many actinobacteria in the studied ecosystem cannot effectively absorb and utilize this type of single carbon or nitrogen source. Moreover, a total of 49 actinobacterial strains were obtained from two media (M9 and M10) supplemented with high contents of multi-salts. Among these isolates, *Streptomyces* (20 strains), *Nesterenkonia* (13 strains) and *Kocuria* (4 strains) were the most abundant genera. Genera *Streptomyces*, *Kocuria*, *Nesterenkonia*, *Nocardiopsis*, *Haloactinobacterium*, *Saccharopolyspora*, *Actinopolyspora*, et al. are often reported as halotolerant or halophilic actinobacteria found in salty desert environment [77]. Herein genera obtained from high-salts media agree with the previous studies on population composition of actinobacteria in different saline or hypersaline habitats.

Desert actinobacteria are recognized versatile sources of biologically active compounds [78,79,80]. In the antibacterial assay, 23 isolates affiliated to 5 genera exhibited antagonistic activity against the tested “ESKAPE” pathogens, in which *Streptomyces* (18 strains) was still the most prominent producer of bioactive metabolites. To gain fast insights into the underlying mechanism of action of these antibacterial strains, a high throughput screening (HTS) model with a dual-reporter assay (pDualrep2) was employed, allowing to early identification of molecules capable of blocking the bacterial translational process or disrupting DNA replication [81,82,83,84,85]. In the present research, secondary metabolites produced by two *Streptomyces* strains, MGa1-3 and MGa2-5, demonstrated the translation inhibitory activity in the pDualrep2 assay, spurring us to prioritize them for further investigation. Notably, crude extracts from most antibacterial-producing strains did not emit any fluorescence in the pDualrep2 system, suggesting that they inhibited bacterial growth by other antibacterial mechanisms. In-depth investigation on bioactive components of these strains should also be carried out in the near future.

Dereplication of the well-known and trivial compounds from complex natural extracts is always a challenging task in novel antibiotics discovery. In this study, chemical profiles of bioactive extracts from three *Streptomyces* strains were analyzed with comprehensive strategies, including bioassay-guided TLC, UPLC-UV-MS/MS, enhanced molecular networking and database dereplication. By search of UPLC-UV-MS/MS data against NPAtlas and StreptomeDB databases in the UNIFI informatics platform, several metabolites affiliated to the family of althiomycin, chloramphenicol and granaticin were identified. The enhanced molecular network was constructed by a powerful tool, namely MolNetEnhancer, which unites the outputs from GNPS molecular networking, MS2LDA substructure discovery, in silico annotation tools (such as NAP or DEREPLICATOR) as well as the automated chemical classifications. Herein, we combined GNPS with NAP annotation in the dereplication process, allowing to annotation of several unknown derivatives of the known antibiotics althiomycin and granaticin. These putative novel compounds will be prioritized for further purification and structure elucidation to ascertain their bioactive structures.

The isolation of althiomycin was firstly described by Yamaguchi et al. from a *Streptomyces* strain 245-Z2 isolated from a soil sample collected in hyogo prefecture, Japan, and the isolate was assigned as a novel *Streptomyces* species, *Streptomyces althioticus* [47]. Strain MGa2-5 showed the maximum identity to the type strain of *S. althioticus*, indicating the possible production of althiomycin by strain MGa2-5. Strain *S. althioticus* MSM3, isolated from the macroalgae collected in Cantabrian Sea produced the macrolide antibiotic desertomycin G with strong antibiotic activity against clinical isolates of *Mycobacterium tuberculosis* [86]. In addition to *S. althioticus*, althiomycin was also found in other *Streptomyces* species such as *S. matensis* [87] and *S. chartreusis* [48]. Members of the myxobacterial genera *Myxococcus* [50] and *Cystobacter* [51], as well as the insect pathogen *Serratia marcescens* [52] were also reported to produce this compound. Althiomycin has a broad-spectrum antibacterial activity, and the 4-methoxy-3-pyrrolin-2-one moiety is a key pharmacophore for its antibacterial activity [88]. The broad antibacterial spectrum, low cytotoxicity and specific selectivity towards prokaryotes make althiomycin as a potentially therapeutic agent. Nevertheless, difficulties in efficient chemical synthesis of althiomycin have hindered further pharmacodynamics investigations [52,89].

The isolate MGa1-3 exhibited the highest 16S rRNA sequence similarity to the type strain of *S. venezuelae*, a pioneer and classical producer of chloramphenicol [90]. Although several soil-derived actinobacteria were reported as producers of chloramphenicol, the biosynthetic pathway has been analyzed mostly in *S. venezuelae* strain. In addition to chloramphenicol, trace amounts of *N*-acetyl, *N*-propionyl and *N*-butyryl amides of *p*-nitrophenylserinol (corynecin I, II and III) were also found in cultural broth of *S. venezuelae* under normal cultural conditions [91]. Higher amounts of corynecins were produced in *S. venezuelae* cultures when the medium was deprived of halogen ions [92], and these substances can also be accumulated in the gene blocked mutants of *S. venezuelae* strain [93]. In our study, chloramphenicol along with corynecin III were detected in culture broth of strain MGa1-3, but corynecin I and II were not detected for some unknown reasons. As one of the most well-documented antibiotics, the clinical usage of chloramphenicol is currently limited due to its major side effects and increasing bacterial resistance. Even so, currently chloramphenicol has still been frequently used as a platform to obtain derivatives with increased potency, because its dichloracetyl moiety can be easily replaced with a variety of other chemical scaffolds, rendering it with potentially greater pharmaceutical properties [94]. Bacteriostatic effect of both althiomycin and chloramphenicol was caused by interfering the action of the peptidyl transferase in the 50S subunit of the bacterial ribosome [95,96,97,98], which is in accordance with the screening result in our dual-reporter pDualrep2 assay.

Strain MGa5-5 shared the highest 16S rRNA sequence similarity with the type strain of *S. lateritius*, which was reported to produce a number of quinone antibiotics of the granatin series [42,43,46]. Fleck et al. characterized granaticin A, dihydrogranaticin A and the methyl ester derivatives from a strain of *S. lateritius* [46], while Martin et al. identified granaticin B, dihydrogranaticin B and granaticinic acid along with their oxides and epoxides from *S. lateritius* ATCC 19913 [42,43]. In the present study, the chemical profile of strain MGa5-5 is similar to the strain of Fleck et al. with several differences. Strain MGa5-5 produced granaticin A and dihydrogranaticin A as the major active metabolites with some minor components of dihydrolated and deacetylated derivatives, but granaticin B or dihydrogranaticin B was undetected therein. Extensive biological activities have been reported for granaticins, including antibacterial, antiprotozoal and cytotoxic activities [41,42,43,44], and they were reported as multi-targeted enzyme inhibitors on both prokaryotes and higher organisms, such as Leucyl-tRNA synthetase [99,100], farnesyltransferase (FTase) [101], inosine 5′-monophosphate dehydrogenase (IMPDH) [102] and cell division cycle kinase (Cdc7) [103]. In recent years, some derivatives of granaticin have been used in pharmaceuticals as an excipient for treating proliferative disease or to treat diseases such as Hartnup syndrome [104]. Therefore, exploring the new derivatives of Granaticin is still of great significance. Meanwhile, the naphthoquinone core confers Granaticin orange to violet colors depending on different pH. Pigments such as melanin, prodigiosin and carotenoids are often regarded with antioxidative and ultraviolet-resistant properties that protect organisms against detrimental effects of ultraviolet radiation [105,106]. Ding et al. [107] identified granaticin A and C as the main active metabolites from a *Streptomyces* strain Sd-31, which was isolated from desert ecosystems in Qinghai–Tibet Plateau. Gurovic et al. [108] reported a series of pigmented antibiotics of granaticin class were identified from a *Streptomyces* sp. SUE01, which was isolated from Extra-Andean Patagonia, an arid region in South America. Since our bioprospection in the arid Mongolia Gobi Desert also manifested a bioactive strain with production of this type of antibiotics, it is worthy to infer that granaticins might have an ecological function to protect microorganisms from damaging UV radiation in arid environments.

## 5. Conclusions

The current study demonstrated the saxaul forest in the Mongolia Gobi Desert harbors an excellent source of taxonomically diverse, culturable actinobacterial strains with potential to produce bioactive metabolites. Combinational strategies of the dual-fluorescent reporter assay, bioassay-guided TLC, UPLC-MS/MS based metabolomic analysis and enhanced molecular networking successfully illuminate the secondary metabolites responsible for antibacterial activities produced by three *Streptomyces* strains. Furthermore, potentially novel secondary metabolites are discovered by in-depth analysis of metabolomic data. We demonstrate that metabolomics profiling and molecular networking are indeed conducive to guide the discovery of novel bioactive candidates. The results in this report not only provide additional information such as microbial diversity and novelty of actinobacteria associated with the Mongolia desert, but also exhibit a practical way to identify known and novel secondary metabolites from actinobacteria isolated from the special environment.

## Data Availability

The 16S rRNA sequences presented in this study are available in GenBank in NCBI (accession numbers: OM943700-OM943745).

## Supplementary Materials

The following supporting information can be downloaded at: https://www.mdpi.com/article/10.3390/microorganisms10050989/s1. Table S1: Compositions of the ten different media used for the isolation of actinobacteria in the study; Table S2: Taxonomic statistics of the isolated 172 actinobacterial strains; Table S3: Antibacterial activity of 23 strains isolated from soil of the saxaul forest in Southern Gobi Desert in Mongolia; Figure S1: Neighbor-joining phylogenetic tree based on 16S rRNA gene sequences of five potential novel strains; Figure S2: UPLC-MS/MS spectra of the putative granaticin A (compound **1**) from strain MGa5-5 and the authentic standard of granaticin A; Figure S3: UPLC-UV-HRMS/MS spectra and identification of bioactive compounds from EA extract of strain MGa2-5; Figure S4: UPLC-UV-HRMS/MS spectra and identification of bioactive compounds from EA extract of strain MGa1-3; Figure S5: UPLC-MS/MS spectra of the putative chloramphenicol (compound **5**) from strain MGa1-3 and the authentic standard of chloramphenicol; Figure S6: UPLC-MS/MS spectra acquired by MS^E^ method and possible fragmentation patterns of putative granaticin compounds (**6**–**10**) from strain MGa5-5; Figure S7: UPLC-MS/MS spectra acquired by MS^E^ method and possible fragmentation patterns of putative chloramphenicol derivatives (**11**–**13**) from strain MGa1-3. Figure S8: UPLC-MS/MS spectra acquired by MS^E^ method and possible fragmentation patterns of putative althiomycin compounds (**14**–**16**) from strain MGa2-5.
