# Supplementary material for: Exploring the Diversity and Antibacterial Potentiality of Cultivable Actinobacteria from the Soil of the Saxaul Forest in Southern Gobi Desert in Mongolia
Source: Microorganisms. 2022 May 9;10(5):989. doi: 10.3390/microorganisms10050989 (PMC9147431; doi:10.3390/microorganisms10050989)

Supplementary Materials

Exploring the Diversity and Antibacterial Potentiality of Cultivable Actinobacteria from the Soil of the Saxaul Forest in Southern Gobi Desert in Mongolia

Shao-Wei Liu [1], Norovsuren Jadambaa [2], Arina A. Nikandrova [3,4], Ilya A. Osterman [3,5,6] and Cheng-Hang Sun [1,*]

[1] Department of Microbial Chemistry and Beijing Key Laboratory of Antimicrobial Agents, Institute of Medicinal Biotechnology, Chinese Academy of Medical Sciences & Peking Union Medical College, Beijing 100050, China; liushaowei3535@163.com
[2] The Institute of Biology, Mongolian Academy of Sciences, Ulaanbaatar 13330, Mongolia; norvo@mail.ru
[3] Center of Life Sciences, Skolkovo Institute of Science and Technology, Moscow 143028, Russia; arinanikandrova@mail.ru (A.A.N.); i.osterman@skoltech.ru (I.A.O.)
[4] Department of Soil Science, Lomonosov Moscow State University, Moscow 119991, Russia
[5] Department of Chemistry, Lomonosov Moscow State University, Moscow 119991, Russia
[6] Genetics and Life Sciences Research Center, Sirius University of Science and Technology, Sochi 354340, Russia
* Correspondence: chenghangsun@hotmail.com

Content

Table S1. Compositions of the ten different media used for the isolation of actinobacteria in the study.
Table S2. Taxonomic statistics of the isolated 172 actinobacterial strains.
Table S3. Antibacterial activity of 23 strains isolated from soil of the saxaul forest in southern Gobi Desert in Mongolia.
Figure S1. Neighbor-joining phylogenetic tree based on 16S rRNA gene sequences of five potential novel strains.
Figure S2. UPLC-MS/MS spectra of the putative granaticin A (compound **1**) from strain MGa5-5 and the authentic standard of granaticin A.
Figure S3. UPLC-UV-HRMS/MS spectra and identification of bioactive compounds from EA extract of strain MGa2-5.
Figure S4. UPLC-UV-HRMS/MS spectra and identification of bioactive compounds from EA extract of strain MGa1-3.
Figure S5. UPLC-MS/MS spectra of the putative chloramphenicol (compound **5**) from strain MGa1-3 and the authentic standard of chloramphenicol.
Figure S6. UPLC-MS/MS spectra acquired by MS^E method and possible fragmentation patterns of putative granaticin compounds (**6-10**) from strain MGa5-5.
Figure S7. UPLC-MS/MS spectra acquired by MS^E method and possible fragmentation patterns of putative chloramphenicol derivatives (**11-13**) from strain MGa1-3.
Figure S8. UPLC-MS/MS spectra acquired by MS^E method and possible fragmentation patterns of putative althiomycin compounds (**14-16**) from strain MGa2-5.

Table S1. Compositions of the ten different media used for the isolation of actinobacteria in the study.

NO.	Name	Composition (In 1.0 L distilled water)
M1	Modified Gauze's NO. 1 synthetic medium	Starch 2.0 g, KNO_3 0.5 g, KCl 1.7 g, $MgSO_4$•$7H_2O$ 0.5 g, Na_2HPO_4 0.5 g, NaCl 0.5g, $CaCO_3$ 0.02 g, $FeSO_4$•$7H_2O$ 0.01 g, Vitamin mixture[a] 1.0 mL, Agar 20.0 g, pH 8.0
M2	ISP 2 medium	Yeast extract 4.0 g, Glucose 4.0 g, Malt extract 5.0 g, Vitamin mixture[a] 1.0 mL, Trace salt[b] 1.0 mL, Agar 20.0 g, pH 8.0
M3	R2A medium	R2A (BD) 18.6 g, Agar 12.0 g, pH 8.0
M4	Modified Cellulose-Casein medium	Cellulose 10.0 g, Casein 0.3 g, K_2HPO_4 0.2 g, $FeSO_4$•$7H_2O$ 0.01 g, $CaCO_3$ 0.02 g, KNO_3 2.0 g, $MgSO_4$•$7H_2O$ 0.05 g, NaCl 10g, Agar 20.0 g, pH 8.0
M5	CMKA medium	Casein acids hydrolysate 0.5 g, Mannitol 1.5g, KNO3 1.0 g, $(NH_4)_2$SO4 2.0 g, K_2HPO_4 0.5g, $CaCO_3$ 0.5g, NaCl 10.0 g, KCl 5.0g, $MgCl_2$ 1.0 g, Agar 20.0 g, pH 8.0
M6	Raffinose-Histidine medium	Raffinose 1.0 g, Histidine 0.1 g, Na_2HPO_4 0.5 g, KCl 1.7g, $MgSO_4$•$7H_2O$ 0.05 g, $FeSO_4$•$7H_2O$ 0.1 g, $CaCO_3$ 0.02 g, Vitamin mixture[a] 1.0 mL, Agar 20.0 g, pH 8.0
M7	Trehalose-Proline medium	Trehalose 5.0 g, L-Proline 1.0 g, (NH4)$_2SO_4$ 1.0 g, $CaCl_2$ 2.0 g, NaCl 1.0 g, K_2HPO_4 1.0 g, $MgSO_4$•$7H_2O$ 1.0 g, Vitamin mixture[a] 1.0 mL, Agar 20.0 g, pH 8.0
M8	Proline medium	L-Proline 5.0 g, Agar 20.0 g, Distilled water 1.0 L, pH 8.0
M9	Casein-Glucose medium	Casein 0.3 g, Glucose 10.0 g, KNO_3 2.0g, $MgSO_4$•$7H_2O$ 0.05 g, K_2HPO_4 2.0 g, $CaCl_2$ 1.0 g, $FeSO_4$•$7H_2O$ 0.01 g, NaCl 50.0 g, KCl 20.0 g, $MgCl_2$•$6H_2O$ 10.0 g, Agar 20.0 g, pH 8.0
M10	Casein-Glucose medium with 16% (w/v) multi-salts	Casein 0.3 g, Glucose 10.0 g, KNO_3 2.0g, $MgSO_4$•$7H_2O$ 0.05 g, K_2HPO_4 2.0 g, $CaCl_2$ 1.0 g, $FeSO_4$•$7H_2O$ 0.01 g, NaCl 100.0 g, KCl 40.0 g, $MgCl_2$•$6H_2O$ 20.0 g, Agar 20.0 g, pH 8.0

Note: [a] Vitamin mixture: thiamine 0.1 g, pyridoxine 0.1 g, riboflavin 0.1g, niacin 0.1 g, biotin 0.1 g, distilled water 100.0 mL.
[b] Trace salt solution: FeSO4•7H2O 0.2 g, MnCl2•4H2O 0.01 g, ZnSO4•7H2O 0.01 g, distilled water 100.0 ml.

Table S2. Taxonomic statistics of the isolated 172 actinobacterial strains.

Taxon			No. of isolates
**Order (7)**	**Family (13)**	**Genus (22)**	
Streptomycetales	*Streptomycetaceae*	*Streptomyces*	73
Micrococcales	*Microbacteriaceae*	*Agromyces*	23
		Labedella	6
		Microbacterium	2
		Agrococcus	1
	Micrococcaceae	*Kocuria*	17
		Nesterenkonia	13
		Arthrobacter	4
		Citricoccus	4
	Promicromonosporaceae	*Isoptericola*	7
		Promicromonospora	2
		Krasilnikoviella	1
	Dermabacteraceae	*Brachybacterium*	2
	Bogoriellaceae	*Georgenia*	1
Mycobacteriales	*Gordoniaceae*	*Gordonia*	4
		Williamsia	1
	Nocardiaceae	*Rhodococcus*	4
	Mycobacteriaceae	*Mycobacterium*	2
Streptosporangiales	*Nocardiopsaceae*	*Nocardiopsis*	2
Propionibacteriales	*Nocardioidaceae*	*Aeromicrobium*	1
Micromonosporales	*Micromonosporaceae*	*Micromonospora*	1
Glycomycetales	*Glycomycetaceae*	*Glycomyces*	1
Total number of actinobacterial isolates			172

Table S3. Antibacterial activity of 23 strains isolated from soil of the saxaul forest in southern Gobi Desert in Mongolia.

**NO.**	**Isolates (GenBank accession NO.)**	**Top-hit taxon (Pairwise similarity)**	**Fractions**[a]	**Activity**[b]											
				E. coli		*P. aeruginosa*		*K. peneumoniae*		*A. baumannii*		*S. aureus*		*E. faecalis*	
				**S**	**R**	**S**	**R**	**S**	**R**	**S**	**R**	**S**	**R**	**S**	**R**
1	MGc3-2 (OM943726)	*Agromyces ramosus* DSM 43045^{T}, 99.5%	E	—	—	—	—	—	—	—	0.9	—	—	—	—
			W	—	—	—	—	—	—	—	—	—	—	—	—
			M	—	—	—	—	—	—	—	—	—	—	—	—
2	MGc3-9 (OM943706)	*Georgenia daeguensis* 2C6-43^{T}, 99.8%	E	—	—	—	—	—	—	—	—	—	—	—	0.8
			W	—	—	—	—	—	—	—	—	—	—	—	—
			M	—	—	—	—	—	—	—	—	—	—	—	1.0
3	MGa2-7 (OM943707)	*Gordonia effusa* IFM 10200^{T}, 99.2%	E	0.7	—	—	—	—	—	—	—	—	—	—	—
			W	—	—	—	—	—	—	—	—	—	—	—	—
			M	—	—	—	—	—	—	—	—	—	—	—	—
4	MGb3-14 (OM943744)	*Gordonia effusa* IFM 10200^{T}, 99.1%	E	0.8	—	—	—	—	—	—	—	—	—	—	—
			W	—	—	—	—	—	—	—	—	—	—	—	—
			M	—	—	—	—	—	—	—	—	—	—	—	—
5	MGb4-1 (OM943714)	*Mycobacterium frederiksbergense* DSM 44346^{T}, 99.7%	E	—	—	—	—	—	—	—	—	—	—	—	—
			W	—	—	—	—	—	—	—	—	0.8	—	—	—
			M	—	—	—	—	—	—		—	—	—	—	—
6	MGa4-10 (OM943727)	*Streptomyces cahuitamycinicus* 13K301^{T}, 99.9%	E	—	—	—	—	—	—	—	—	0.9	—	—	—
			W	—	—	—	—	—	—	—	—	—	—	—	—
			M	—	—	—	—	—	—	—	—	—	—	—	—
7	MGa6-1 (OM943728)	*Streptomyces flavovariabilis* NRRL B-16367^{T}, 99.3%	E	—	—	—	—	—	—	—	—	—	—	1.0	1.1
			W	—	—	—	—	—	—	—	—	—	—	—	—

			M	—	—	—	—	—	—	—	—	—	—	—	—
8	MGa4-1 (OM943729)	*Streptomyces fradiae* DSM 40063^T, 99.5%	E	—	0.7	—	—	—	—	1.5	—	—	—	—	—
			W	—	—	—	—	—	—	—	—	—	—	—	—
			M	—	—	—	—	—	—	—	—	—	—	—	—
9	MGa3-5 (OM943730)	*Streptomyces iakyrus* NRRL ISP-5482^T, 100.0%	E	—	—	—	—	—	—	—	—	1.0	1.3	—	1.3
			W	—	—	—	—	—	—	—	—	—	—	—	—
			M	—	—	—	—	—	—	—	—	—	—	—	—
10	MGa9-14 (OM943731)	*Streptomyces parvus* NBRC 3388^T, 100.0%	E	0.8	0.7	—	—	1.1	—	—	—	0.8	1.4	0.9	1.2
			W	—	—	—	—	—	—	—	—	—	—	—	—
			M	—	—	—	—	—	—	—	—	—	—	—	—
11	MGc7-5 (OM943732)	*Streptomyces plumbiresistens* CCNWHX 13-160^T, 99.9%	E	—	—	—	—	—	—	—	—	0.9	0.9	0.9	1.0
			W	—	—	—	—	—	—	—	—	—	—	—	—
			M	—	—	—	—	—	—	—	—	0.7	0.9	—	—
12	MGb9-6 (OM943733)	*Streptomyces pluricolorescens* NBRC 12808^T, 98.8%	E	—	—	—	—	—	—	—	—	—	—	—	—
			W	1.7	1.3	—	—	1.1	—	—	0.7	0.7	—	—	—
			M	—	—	—	—	—	—	—	—	—	—	—	—
13	MGs9-7 (OM943734)	*Streptomyces purpurascens* NBRC 13077^T, 99.0%	E	—	—	—	—	—	—	—	—	1.0	0.9	1.3	1.0
			W	—	—	—	—	—	—	—	—	—	—	—	—
			M	—	—	—	—	—	—	—	—	—	—	—	—
14	MGa9-13 (OM943735)	*Streptomyces rectiviolaceus* NRRL B-16374^T, 99.9%	E	—	—	—	—	—	—	—	—	1.2	0.8	0.9	—
			W	—	—	—	—	0.6	—	—	—	—	—	—	—
			M	—	—	—	—	—	—	—	—	—	—	—	—
15	MGc9-8 (OM943736)	*Streptomyces sparsus* YIM 90018^T, 99.8%	E	—	—	—	—	—	—	—	0.8	1.0	1.2	—	—
			W	—	—	—	—	—	—	—	—	—	—	—	—
			M	—	—	—	—	—	—	—	—	—	—	—	—
16	MGc3-10	*Streptomyces sparsus* YIM	E	—	—	—	—	—	—	—	—	—	—	—	—

	(OM943737)	90018^{T}, 99.8%	W	—	—	—	—	—	—	—	—	0.8	0.7	—	—
			M	—	—	—	—	—	—	—	—	—	—	—	—
17	MGb9-13	*Streptomyces sparsus* YIM	E	—	—	—	—	—	—	—	—	0.7	—	—	2.8
	(OM943738)	90018^{T}, 99.8%	W	—	—	—	—	—	—	—	—	—	—	1.5	1.8
			M	—	—	—	—	—	—	—	—	—	1.0	—	1.0
18	MGc5-5	*Streptomyces tuirus* NBRC	E	—	—	—	—	—	—	—	—	0.9	0.8	1.1	1.5
	(OM943739)	15617^{T}, 99.5%	W	—	—	—	—	—	—	—	—	—	—	—	—
			M	—	—	—	—	—	—	—	—	—	—	—	—
19	MGa5-5	*Streptomyces lateritius* LMG	E	—	—	—	—	—	0.7	0.7	1.2	1.4	2.3	0.9	2.0
	(OM943740)	19372^{T}, 99.7%	W	—	—	—	—	—	—	—	0.7	1.2	1.3	—	—
			M	—	—	—	—	—	—	—	—	—	—	—	—
20	MGc2-5	*Streptomyces huasconensis*	E	—	—	—	—	—	—	—	—	1.8	—	—	—
	(OM943741)	HST28^{T}, 99.5%	W	—	—	—	—	—	—	—	—	1.2	1.5	—	—
			M	—	—	—	—	—	—	—	—	1.5	1.8	—	—
21	MGa1-3	*Streptomyces venezuelae* ATCC	E	1.8	1.6	1.7	—	2.0	1.5	1.2	1.3	2.0	0.9	1.3	2.1
	(OM943745)	10712^{T}, 99.7%	W	—	—	—	—	—	—	—	—	—	—	—	—
			M	—	—	—	—	0.9	—	—	—	—	—	—	—
22	MGc5-6	*Streptomyces coeruleoprunus*	E	—	—	—	—	1.1	—	—	—	0.9	—	—	—
	(OM943742)	NBRC 15400^{T}, 99.6%	W	—	—	—	—	—	—	—	—	—	—	—	—
			M	—	—	—	—	—	—	—	—	—	—	—	—
23	MGa2-5	*Streptomyces althioticus* NRRL	E	—	—	—	—	2.0	—	—	—	0.8	1.3	0.7	1.4
	(OM943743)	B-3981^{T}, 98.8%	W	—	—	—	—	—	—	—	—	—	—	—	—
			M	—	—	—	—	—	—	—	—	—	—	—	—

[a] E: crude sample extracted with ethyl acetate; M: crude sample from mycelium; W: crude sample from water layer.

[b] The diameters of the inhibition zones: cm; —, no inhibition.

Figure S1. Neighbor-joining phylogenetic tree based on 16S rRNA gene sequences of five potential novel strains. Numbers at nodes indicate the level of bootstrap support (> 50%) based on 1000 replications. Bar, 2 nt substitutions per 100 nt.

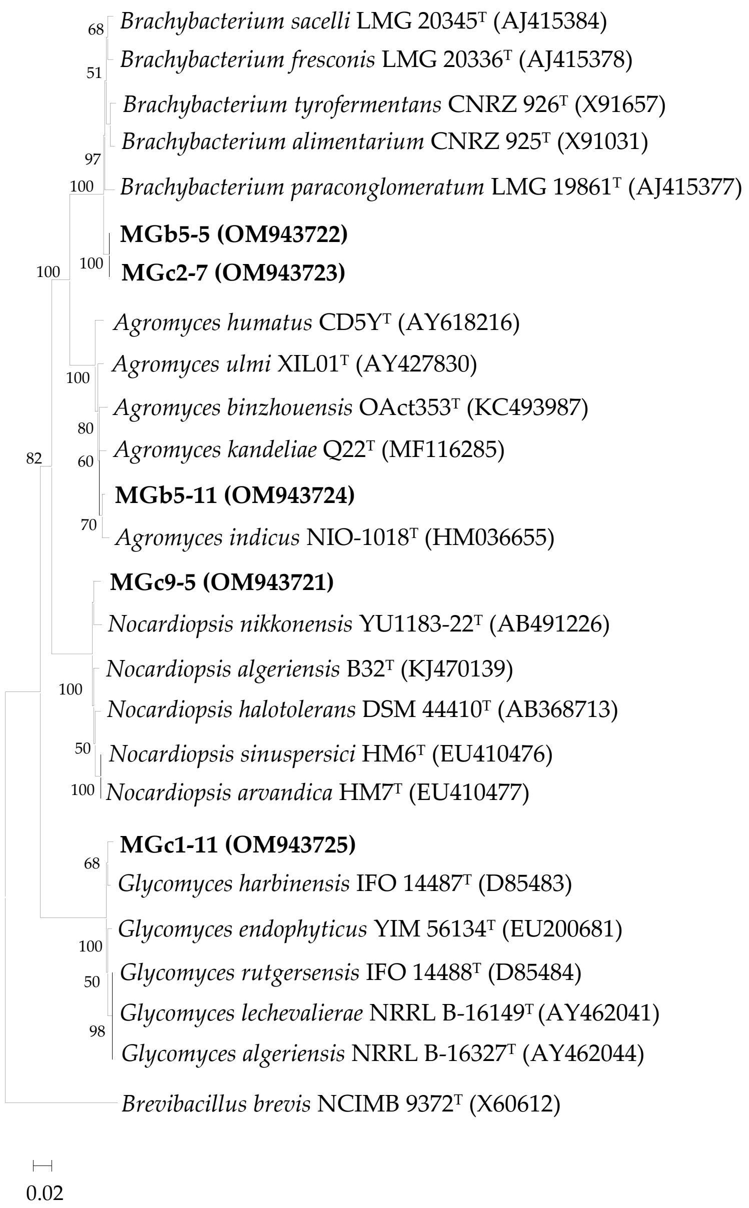

Figure S2. UPLC-MS/MS spectra of the putative granaticin A (compound **1**) from strain MGa5-5 and the authentic standard of granaticin A. (**A**) TIC plots of the TLC band (R_f 0.71-0.79) from the ethyl acetate of cultural broth of strain MGa5-5 and authentic granaticin A. (**B**) MS/MS spectra of the putative granaticin A (**1**) and authentic granaticin A.

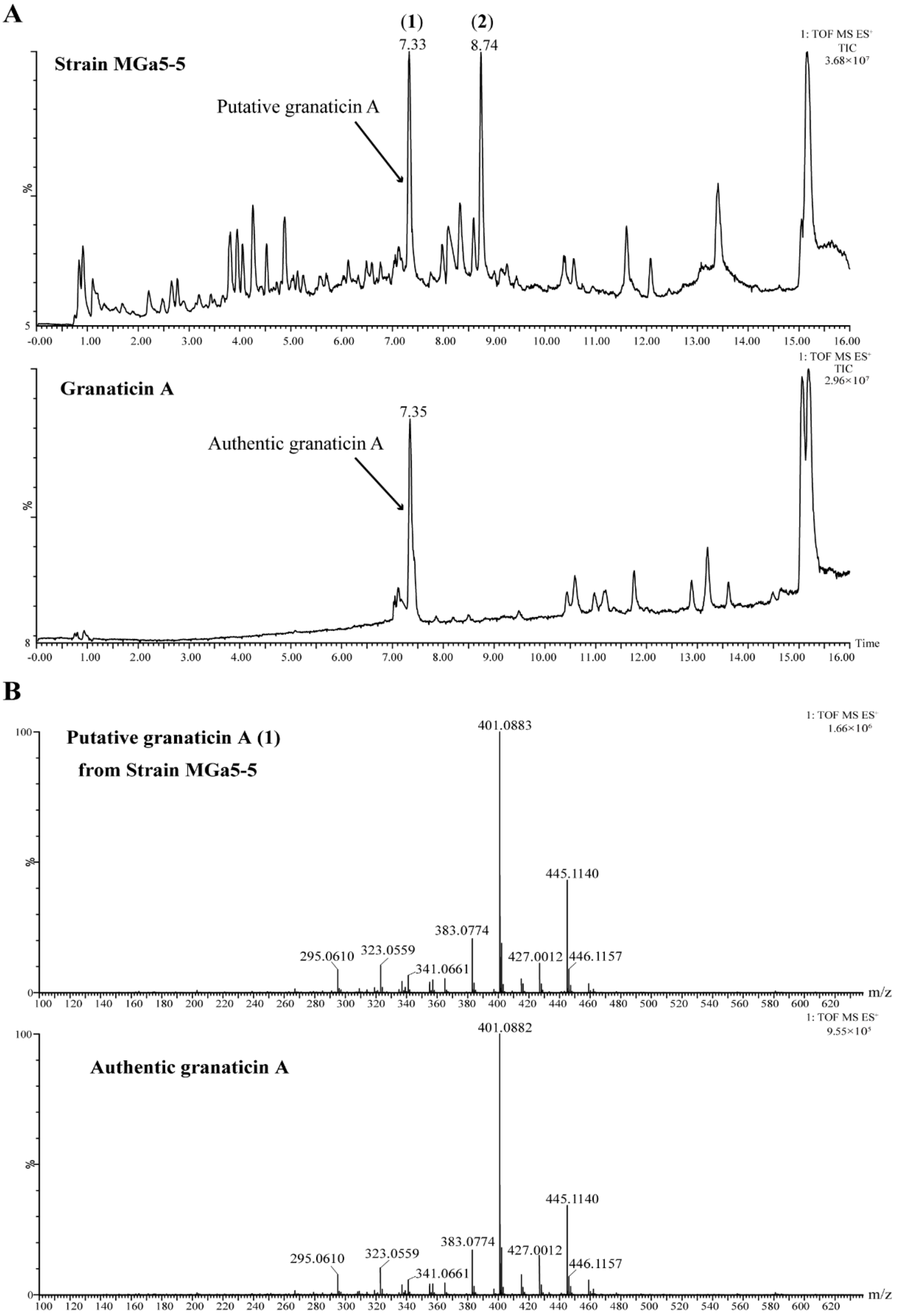

Figure S3. UPLC-UV-HRMS/MS spectra and identification of bioactive compounds from EA extract of strain MGa2-5. (**A**) UPLC-UV chromatogram at 254 nm of the bioactive TLC band (R_f 0.62) extracted from strain MGa2-5, and one major compound (peak **3**) was labelled in the UPLC spectrum. (**B**) TIC plot of the bioactive TLC band (R_f 0.62) extracted from strain MGa2-5. (**C**) MS/MS spectrum and the annotated fragmentation pattern of peak **3** (althiomycin).

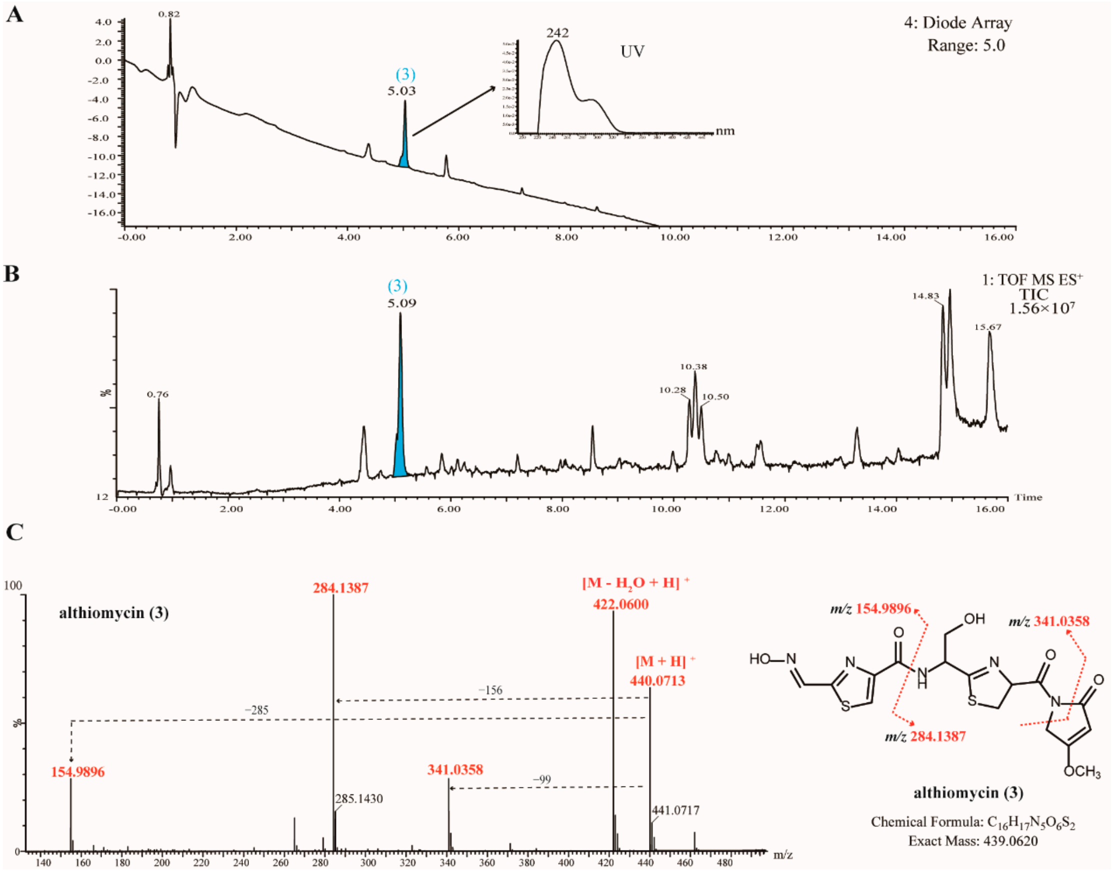

Figure S4. UPLC-UV-HRMS/MS spectra and identification of bioactive compounds from EA extract of strain MGa1-3. (**A**) UPLC-UV chromatogram at 280 nm of the bioactive TLC band (R_f 0.63) extracted from strain MGa1-3, and two major compounds (peak **4** and **5**) were labelled in the UPLC spectrum. (**B**) TIC plot of the bioactive TLC band (R_f 0.63) extracted from strain MGa1-3. (**C**) MS/MS spectra and the annotated fragmentation patterns of peak **4** (corynecin III) and **5** (chloramphenicol).

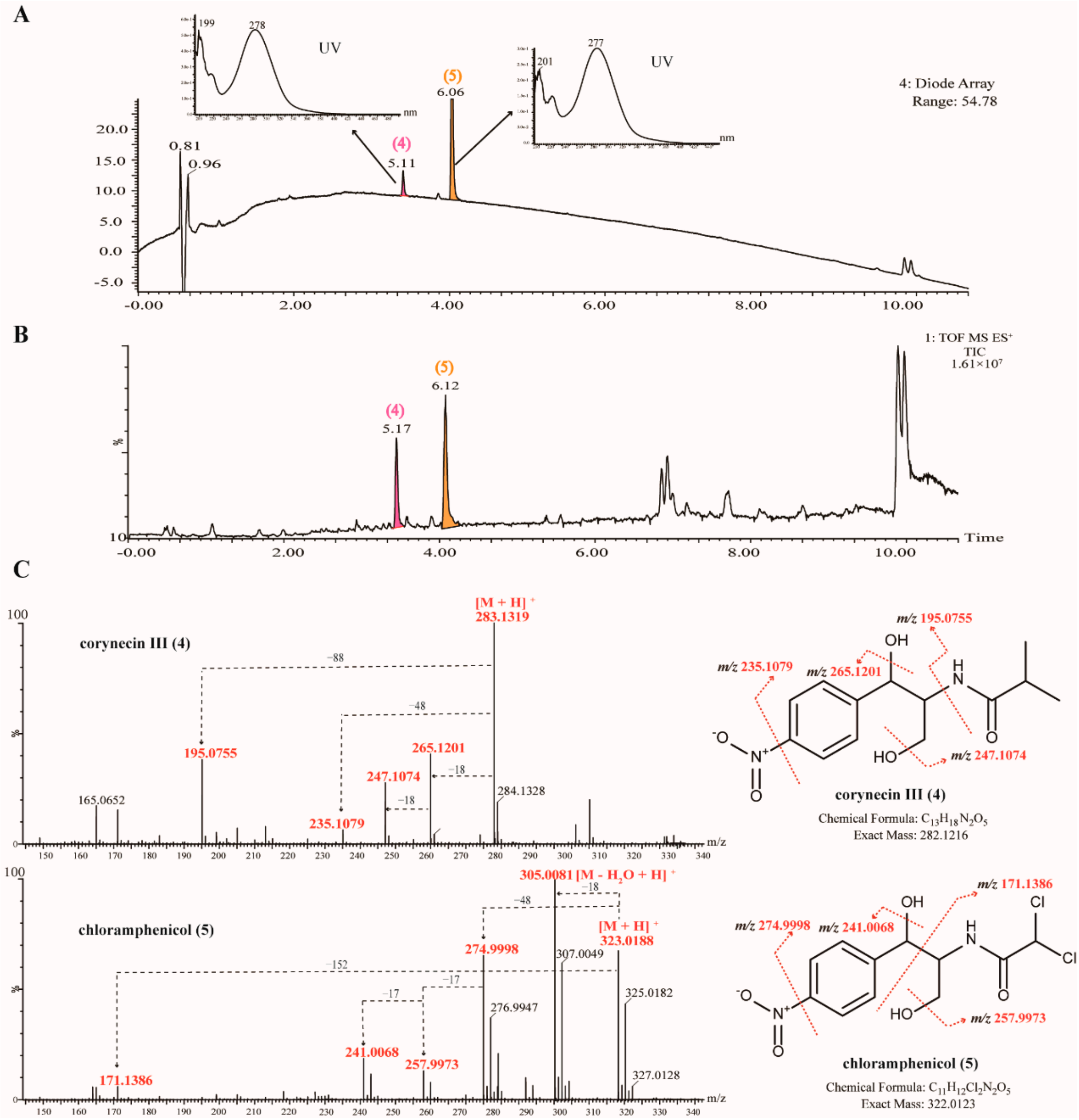

Figure S5. UPLC-MS/MS spectra of the putative chloramphenicol (compound **5**) from strain MGa1-3 and the authentic standard of chloramphenicol. (**A**) TIC plots of the TLC band (R_f 0.63) from the ethyl acetate of cultural broth of strain MGa1-3 and authentic chloramphenicol. (**B**) MS/MS spectra of the putative chloramphenicol (**5**) and authentic chloramphenicol.

A

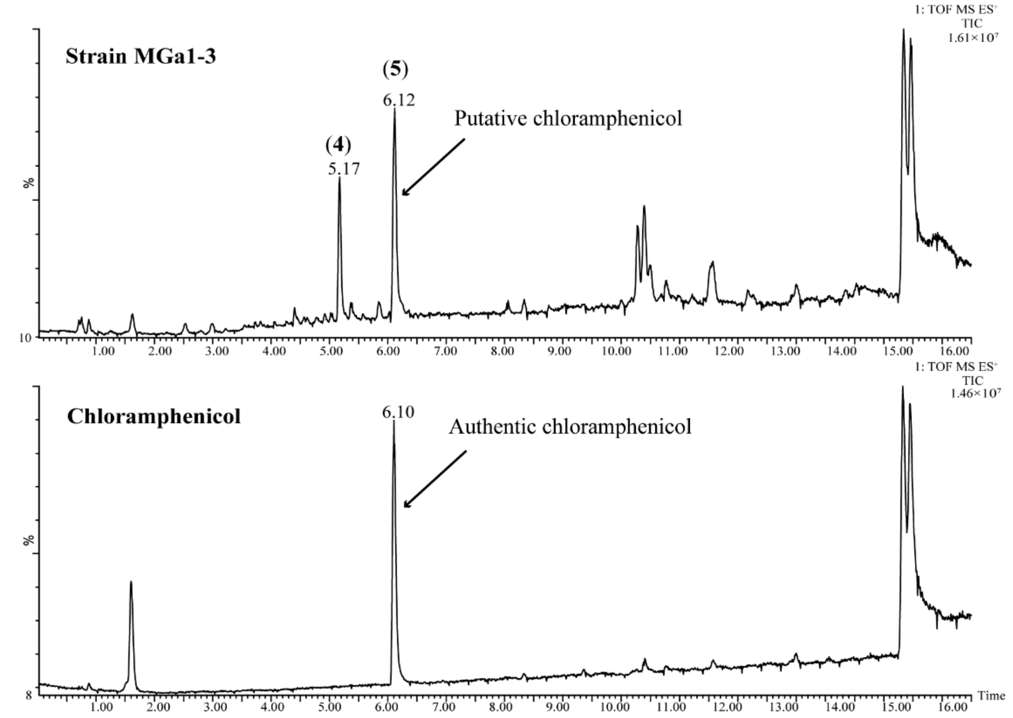

B

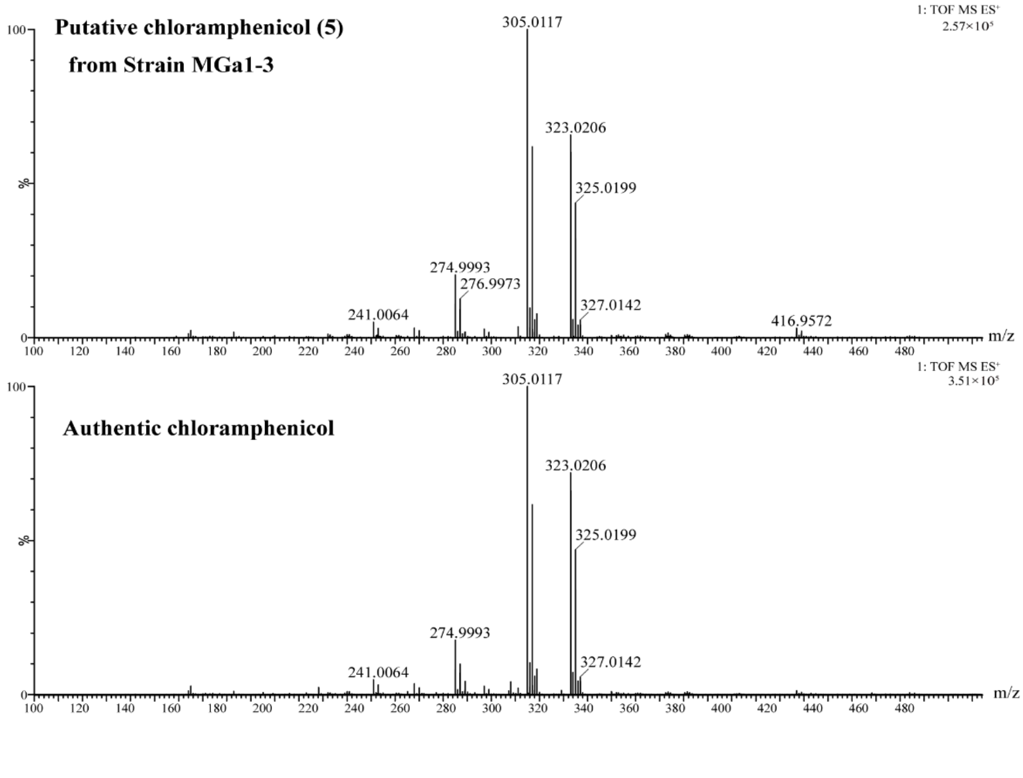

Figure S6. UPLC-MS/MS spectra acquired by MSE method and possible fragmentation patterns of putative granaticin compounds (**6-10**) from strain MGa5-5. (**A**) TIC plot of the ethyl acetate extract from the cultural broth of strain MGa5-5. (**B**) MS/MS spectra of five putative granaticin compounds **6-10** and possible fragmentation patterns of compounds **6-8.**

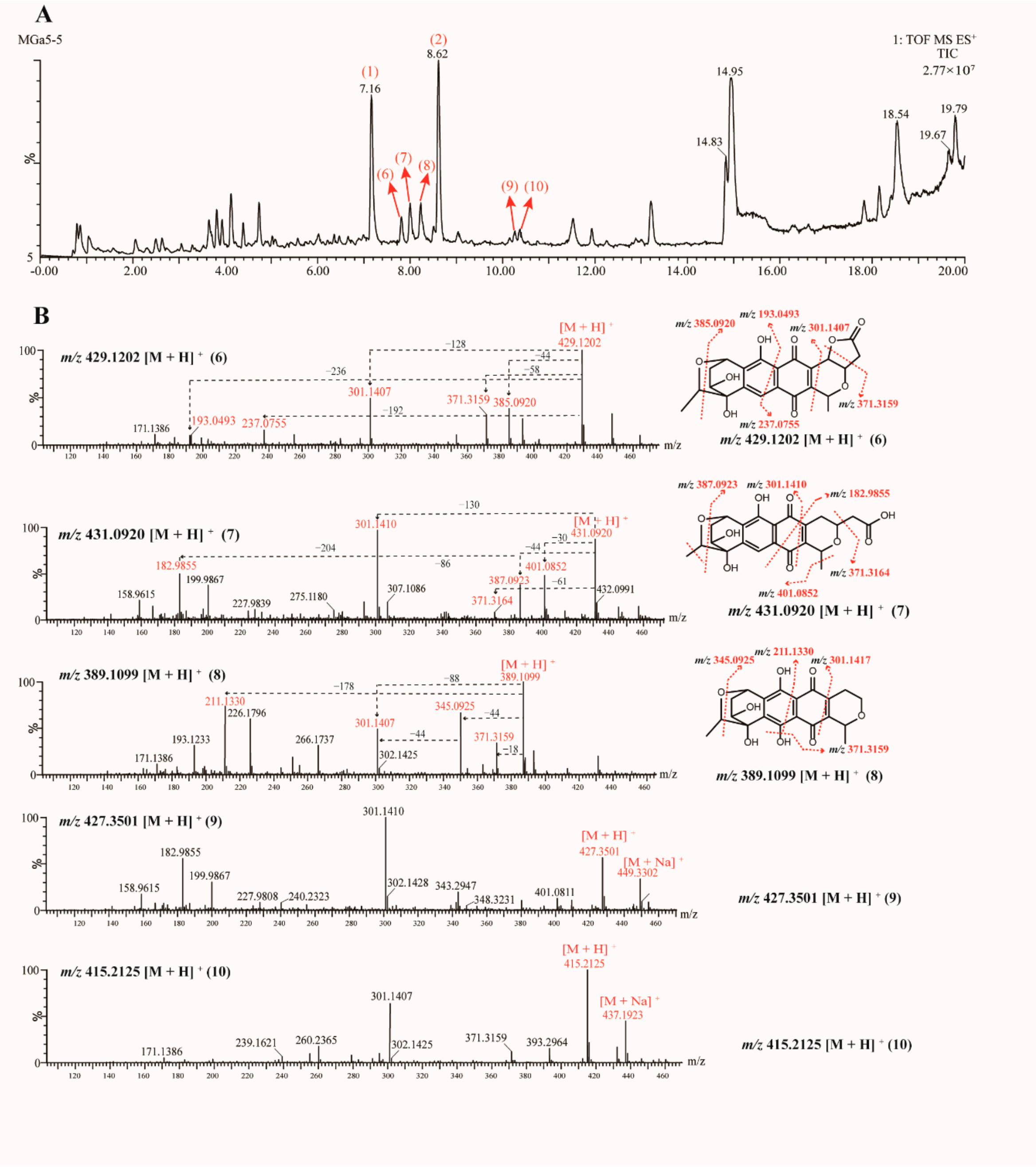

Figure S7. UPLC-MS/MS spectra acquired by MS^E method and possible fragmentation patterns of putative chloramphenicol derivatives (**11-13**) from strain MGa1-3. (**A**) TIC plot of the ethyl acetate extract from the cultural broth of strain MGa1-3. (**B**) MS/MS spectra and possible fragmentation patterns of three putative chloramphenicol derivatives **11-13**.

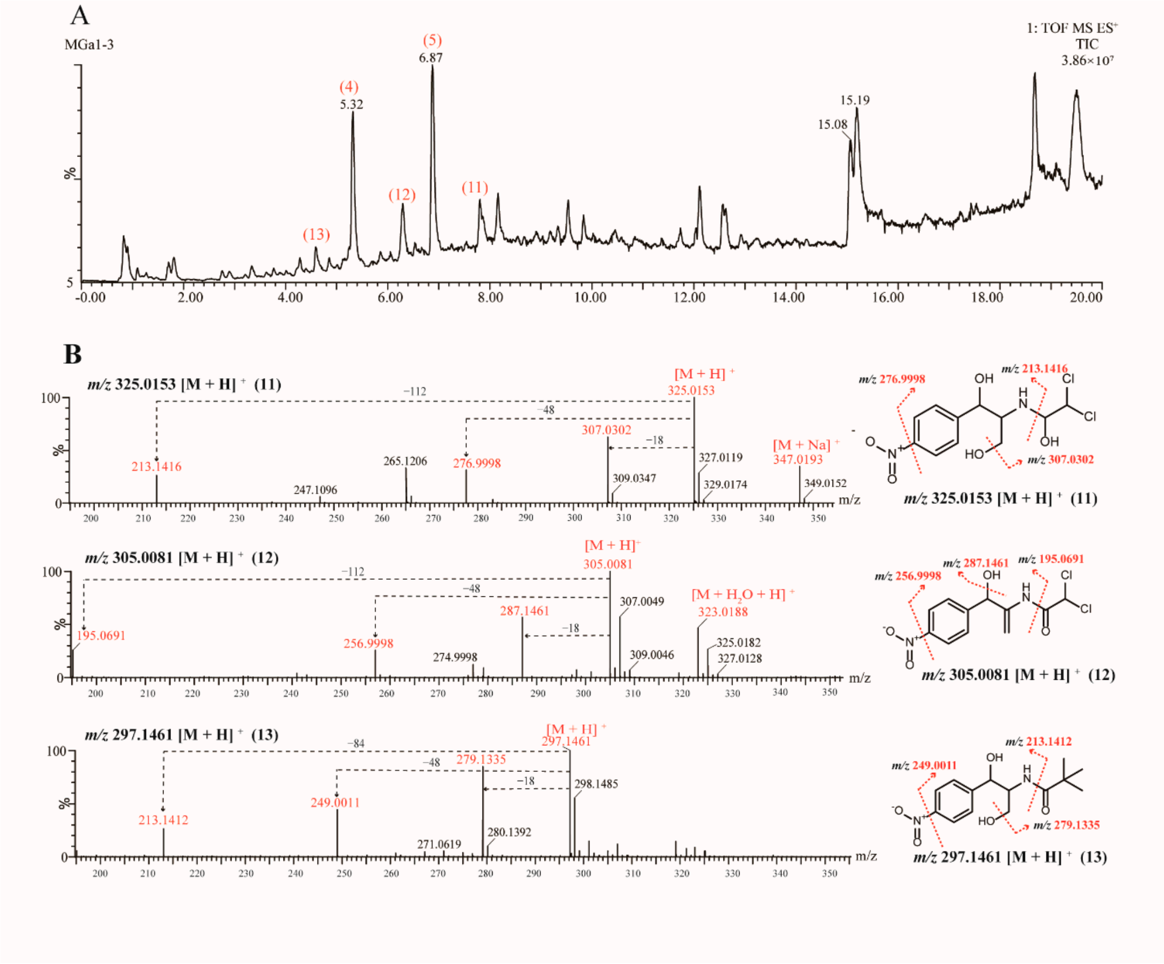

Figure S8. UPLC-MS/MS spectra acquired by MSE method and possible fragmentation patterns of putative althiomycin compounds (**14-16**) from strain MGa2-5. (**A**) TIC plot of the ethyl acetate extract from the cultural broth of strain MGa2-5. (**B**) MS/MS spectra of three putative althiomycin compounds **14-16** and possible fragmentation patterns of compounds **14-15**.

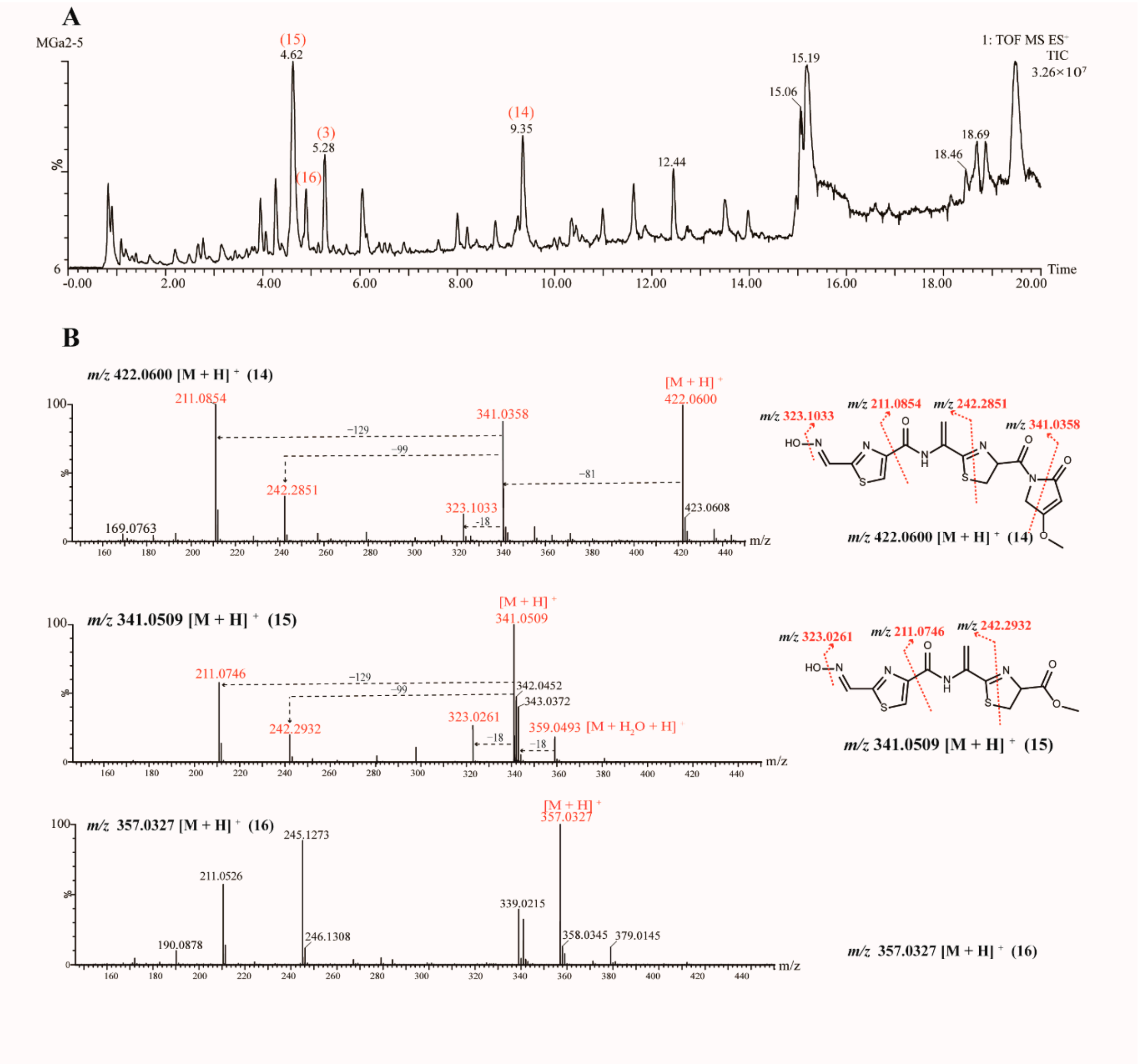

Supplement: Supplementary file 1 [file microorganisms-10-00989-s001.zip › microorganisms-1670913-supplementary.pdf]
